# Polyphagy and diversification in tussock moths: Support for the oscillation hypothesis from extreme generalists

**DOI:** 10.1002/ece3.3350

**Published:** 2017-08-30

**Authors:** Houshuai Wang, Jeremy D. Holloway, Niklas Janz, Mariana P. Braga, Niklas Wahlberg, Min Wang, Sören Nylin

**Affiliations:** ^1^ Department of Entomology South China Agricultural University Guangzhou China; ^2^ Department of Life Sciences Natural History Museum London UK; ^3^ Department of Zoology Stockholm University Stockholm Sweden; ^4^ Department of Biology Laboratory of Genetics University of Turku Turku Finland; ^5^ Department of Biology Lund University Lund Sweden

**Keywords:** host plant range, Lymantriinae, Nymphalidae, plasticity, speciation

## Abstract

Theory on plasticity driving speciation, as applied to insect–plant interactions (the oscillation hypothesis), predicts more species in clades with higher diversity of host use, all else being equal. Previous support comes mainly from specialized herbivores such as butterflies, and plasticity theory suggests that there may be an upper host range limit where host diversity no longer promotes diversification. The tussock moths (Erebidae: Lymantriinae) are known for extreme levels of polyphagy. We demonstrate that this system is also very different from butterflies in terms of phylogenetic signal for polyphagy and for use of specific host orders. Yet we found support for the generality of the oscillation hypothesis, in that clades with higher diversity of host use were found to contain more species. These clades also consistently contained the most polyphagous single species. Comparing host use in Lymantriinae with related taxa shows that the taxon indeed stands out in terms of the frequency of polyphagous species. Comparative evidence suggests that this is most probably due to its nonfeeding adults, with polyphagy being part of a resulting life history syndrome. Our results indicate that even high levels of plasticity can drive diversification, at least when the levels oscillate over time.

## 1 INTRODUCTION

It is generally assumed that there is a causal link between the extreme diversity of phytophagous insects and the diversity of their primary food source, the angiosperm plants (Janz, 2011; Mitter, Farrel, & Wiegmann, 1988; Wahlberg, Wheat, & Pena, 2013). The study of the evolutionary insect–plant interactions that might have caused such patterns has to some extent been dominated by investigations on butterflies and their host plants, ever since the seminal paper on co‐evolution by Ehrlich and Raven (1964). The findings from these studies have provided evidence that butterfly diversification can have been promoted either because of host shifts followed by radiation of new species (Fordyce, 2010; Wheat et al., 2007), as originally suggested by Ehrlich and Raven (1964), or because of diversification associated with transient periods of polyphagy followed by respecialization and speciation (the “oscillation hypothesis of diversification” (Janz & Nylin, 2008; Nylin, Slove, & Janz, 2014), or a combination of both processes. The role of co‐evolution (in the strict sense of reciprocal adaptations in butterflies and plants; Janzen, 1980) is still unclear, but there are indications of macroevolutionary patterns of co‐evolution in pierid butterflies and their host plants, with “key innovations” promoting diversification in both lineages (Edger et al., 2015). More generally, it is likely that diffuse co‐evolutionary processes are often involved when adaptations and counter‐adaptations evolve in local populations of insects and plants (Singer & McBride, 2012; Thompson, 1999).

Evidence for the oscillation hypothesis comes primarily from nymphalid butterflies, where clades with higher diversity of host use than their sister clades tend to contain more species (Janz, Nylin, & Wahlberg, 2006; Nylin & Wahlberg, 2008). Recent studies on nymphalid butterflies using other methods have instead observed patterns seen by the authors as evidence against the oscillation hypothesis of diversification, which thus remains controversial (Hamm & Fordyce, 2015; Hardy & Otto, 2014), but see Discussion and Janz, Braga, Wahlberg, and Nylin (2016).

In butterflies, strong polyphagy is rare and has an apical position in the phylogenies, with low phylogenetic signal (Nylin et al., 2014), indicative of its transient nature in the studied taxa. The taxonomic identity of host use at the level of plant orders is in contrast very conservative, with clades often preferring a given order for many millions of years (Fordyce, 2010; Janz, Nyblom, & Nylin, 2001; Nylin & Wahlberg, 2008; Nylin et al., 2014; Scriber, 2010; Wheat et al., 2007). It is of great interest to study whether evolutionary patterns consistent with the oscillation hypothesis of diversification can be found also in insects with much higher general levels of polyphagy, such as some moths. This is because the hypothesis can be seen as an example of the general theory that phenotypic plasticity can drive evolutionary change and diversification by exposing induced phenotypes to selection, resulting in a process of genetic accommodation that under some circumstances can promote speciation (Nylin & Janz, 2009; Pfennig et al., 2010; West‐Eberhard, 2003).

The oscillation hypothesis of diversification suggests that if speciation in phytophagous insects is promoted by specialization on different host plants (e.g., via “host races”), this process would “run out of fuel” in a lineage if there are not also evolutionary episodes when host ranges become wider, that is, polyphagy (Janz & Nylin, 2008;). Thus oscillations in host range are necessary to produce high speciosity as a general pattern in phytophagous insects. Furthermore, even if shifts to novel host plants are the most important factor in such diversification processes, as suggested by, for example, Ehrlich and Raven (1964), such shifts cannot be instantaneous. Rather, there must be a period of relative polyphagy when both the ancestral and the novel hosts (and most likely other hosts as well) are used (Janz et al., 2006; Nylin & Wahlberg, 2008). As hosts vary in their characteristics, particularly when it comes to chemical composition, phenotypic plasticity is very likely needed for a polyphagous species to be able to cope with its different hosts. There are several aspects of plasticity involved (Nylin & Janz, 2009), but the one most generally linked to the overall theory of developmental plasticity promoting diversification is the plastic patterns of gene expression and enzyme dynamics commonly seen within populations of polyphagous insect species in response to different hosts, with clear links to the ability to digest, detoxify, and metabolize these varying hosts (e.g., Celorio‐Mancera et al., 2013, 2016; Christodoulides et al., 2017; Lazarevic et al., 2017; Mathers et al., 2017; Roy et al., 2016; Schweizer, Heidel‐Fischer, Vogel, & Reymond, 2017).

At the same time, theory suggests that the precise form of plasticity matters greatly for whether it can promote diversification or not (Ghalambor, Mckay, Carroll, & Reznick, 2007), and indeed too high levels of plasticity may instead reduce the likelihood of genetic change (Price, Qvarnstrom, & Irwin, 2003). Studying moth taxa with ubiquitous and very high degrees of polyphagy is thus a strong test of the generality of the oscillation hypothesis for phytophagous insects. A further opportunity presented by studying taxa where polyphagy is less transient than in butterflies would be the possibility of investigating whether polyphagy at the species level can here be seen to be correlated with combined diversity of host use in higher taxa, a pattern that can only be observed as a tendency in the butterfly studies because of the strong tendency for (re‐)specialization (Nylin et al., 2014; Weingartner, Wahlberg, & Nylin, 2006).

**Figure 1:**
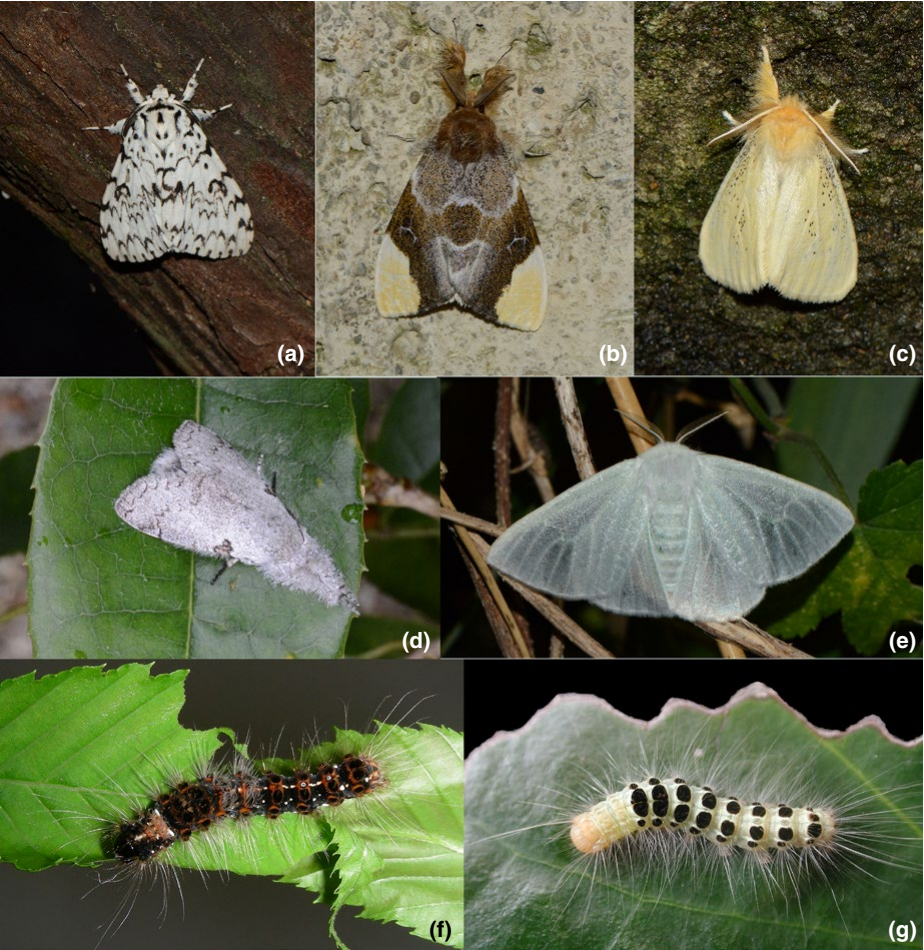
Examples of tussock moths (subfamily Lymantriinae). (a–e): adults. (a): *Lymantria similis* (tribe Lymantriini) (b). *Pida minensis* (Locharnini) (c): *Euproctis conistica* (Nygmiini). (d): *Calliteara contexta* (Orgyiini) *E. Arctornis* sp.(Nygmiini) (f–g): larvae. (f): *Artaxa angulate* (Nygmiini). (g): *Arna bicostata* (Nygmiini). Photo: Houshuai Wang

Studies on moths have however been hindered by the lack of phylogenetic information and by the corresponding taxonomic uncertainty regarding the assignment of host records. The tussock moths (Erebidae: Lymantriinae; Figure 1) are an insect group of predominantly arboreal defoliators, with high levels of polyphagy (Ferguson, 1978; Holloway, Bradley, & Carter, 1987), and about 2,500 described species. Many tussock moths are furthermore serious pest species (Chao, 2003; Schaefer, 1989). Here, we make use of recent advances regarding the phylogeny of Lymantriinae (Wang et al. 2015) as well as the higher‐level patterns in Erebidae (Zahiri et al., 2012) to test the generality of the oscillation hypothesis under high levels of polyphagy—in a system where the phylogenetic signal for polyphagy and for use of specific host orders is very different from what is seen in butterflies. Comparisons with nymphalid butterflies are included to illustrate these differences. We also explore potential reasons for the extreme polyphagy and the associated tendency to produce economically important pests in the tussock moths.

## 2 MATERIALS AND METHODS

### 2.1 Taxon sampling of lymantriinae

Because of the remaining phylogenetic uncertainty in the subfamily, we focused on the around 55 genus‐level taxa included in the recent major phylogenetic investigation by Wang et al. (Wang et al. 2015), representing all the seven recognized tribes of the subfamily and sampled from all major biogeographical regions. There are in total about 360 described genera of tussock moths, but many are monotypic and have been or may eventually become synonymized with other genera as knowledge of the global fauna improves. On the other hand, some traditionally large genera such as *Euproctis* (see below) are proving to be divisible into several distinct generic concepts, a process still in progress. For these reasons, the number of species included in the analyses of species richness actually covers about half of the described species. Still, it must be acknowledged that this study can only be preliminary in this respect.

### 2.2 Lymantriinae host plant data

We searched the literature and online databases (for specific references, see the supporting information) for host records at the species level of the moths, noting the host plant family and order (cf. Table S1). The Angiosperm Phylogeny Website (Stevens, 2001) was used to assign host plant records to currently recognized plant families and orders. It should be noted that there are undoubtedly some host errors present in the material, as well as many unreported host taxa. Moreover, diversity of host use in a clade sometimes must be estimated from only a small portion of the described species, those with host plant records. Our strategy was to investigate whether the signal from the patterns predicted by the oscillation hypothesis can still be seen above this “noise” in the data, and despite the less than complete taxon sampling. The species‐level information was subsequently collated into a table over host use in genus‐level taxa (Table S2), including also estimates of the number of species in each taxon.

### 2.3 Phylogenetic signal

We investigated the phylogenetic signal (i.e., the tendency for related species to resemble each other more than randomly drawn species) for polyphagy and for the use of specific host orders (the eleven most commonly used hosts in Lymantriinae and in Nymphalidae, respectively). Phylogenetic signal is most often investigated using methods which basically compare the evolution of traits with some form of a random walk model, for example, Pagel's lambda (Pagel, 1999). As these commonly used methods were designed for analysis of continuous traits rather than the binary traits studied here, we instead chose to use transition rates between states which are estimated during ancestral character estimation in the package *ape* (Paradis, Claude, & Strimmer, 2004). Low transition rates suggest high phylogenetic signal, and we tested the significance in comparison with 1,000 permutations of the data where the trait values were randomly shuffled on the tree and transition rates recalculated.

### 2.4 Lymantriinae species numbers

Given that there is no modern global catalogue for Lymantriinae, we first estimated a provisional species number for each genus‐level taxon using the online Lepidoptera database of Savela (2014), as well as other information and online sources such as the Global Lepidoptera Names Index (Beccaloni, Scoble, Kitching, & Simonsen, 2005). The species numbers were further revised based on some recent reviews of classification (e.g., Holloway, 1999; Chao, 2003; Schintlmeister, 2004; Pogue & Schaefer, 2007; Kishida, 2011; Speidel & Witt, 2011; Wang, Wang, & Fan, 2011; Wang et al. 2015). According to the phylogenetic investigation of Wang et al. (2015) and the distribution of type species, we restricted *Dasychira* to the Nearctic region, and *Aroa* to the African region, respectively. Both genera are large and complex taxa, and are in urgent need of further systematic revisions. For the “*Euproctis”* complex, found to be nonmonophyletic in the lymantriine phylogeny of Wang et al. (2015), we treated them as several distinct genus‐level clades to assess the species number separately (see Tables S1–S2). Three clades named “*Euproctis*”_1‐3, phylogenetically positioned distantly from the type species are recognized, whereas other “*Euproctis*” species were provisionally included in either a strict‐sense *Euproctis*, when they had positions close to the type species *E. chrysorrhoea*, or in *Toxoproctis* when they were closer to *T. croceola*. The combined estimates of species numbers cover about half of the around 2,500 described species in the subfamily.

### 2.5 Lymantriinae host plant use and diversification

The evolution of host plant use in the subfamily was reconstructed by character optimization using parsimony as implemented in Mesquite (Maddison & Maddison, 2014). First, the characters “3+ orders” and “7+ orders” were traced on the Lymantriinae phylogeny of Wang et al. (2015) for the sake of comparison with the patterns of polyphagy observed in earlier butterfly studies. These are binary characters showing whether *any* species in a given genus‐level taxon have been reported to feed on three or more host orders, or seven or more orders, respectively. Second, the total number of host orders used by the genus‐level taxa was traced on the phylogeny as a continuous character. In this case, a high number does not necessarily indicate current polyphagy, but rather diversity of host use in the clade presumed to reflect past episodes of polyphagy leading to colonizations of new taxa. Third, the use as larval host plants of the ten host orders eaten by the highest number of genus‐level taxa was traced on the phylogeny.

The character optimization and tracing of host diversity as a continuous character visually aided the selection of “sister‐group contrasts.” The contrasted clades may not always turn out to be sister taxa in the strict sense, due to the incomplete taxon sampling and incomplete phylogenetic knowledge, but in some case, they will be, that is, they are “putative” sister clades. We tested the prediction that clades with more diverse host use will tend to contain more species than sister clades utilizing fewer host orders. All contrasts were between putative sister taxa (in the sense given above) and were phylogenetically independent.

Contrasts were constructed in two ways: (1) genus‐level sister taxa were contrasted, regardless of the strength of difference in host diversity, or (2) only strong contrasts were used. In the latter case, with few exceptions the contrasts were also at the taxonomic level of putative sister genera differing in host diversity, but in a few cases, we constructed the contrasts differently. This was done when it was necessary to avoid contrasts with small differences in host range, as they are the ones most sensitive to the quality of the host plant information. We selected a minimum host range difference of three plant orders as the criterion for choice of contrasts, combining genus‐level taxa and their host ranges until this difference was seen.

In order to investigate to what extent the combined diversity of host use also measures actual polyphagy, we also noted which clade in each contrast that contained the most polyphagous single species.

A nonparametric sign test was used to investigate the statistical significance of the results, that is, whether there were more contrasts in the predicted direction (henceforth referred to as a positive contrast) than expected from chance. We also performed the parametric test suggested by Arnqvist, Edvardsson, Friberg, and Nilsson (2000), involving a paired *t* test investigating whether the average of the logarithms of the relative species number in the contrasts is significantly above zero. Positive contrasts will have positive values of this logarithmic measure, as the relative species numbers (No. of species in clade with higher host diversity/No. of species in the clade with lower host diversity) will be above 1, whereas negative contrasts will give rise to negative logarithms.

In order to make full use of all contrasts in the tree, not just apical pairs of putative sister taxa, we also performed a phylogenetic generalized least squares (PGLS) regression (Martins & Hansen, 1997) between host diversity and species richness of genus‐level taxa. PGLS models also include the λ parameter which estimates the codependence of data points due to shared evolutionary history (Freckleton, Harvey, & Pagel, 2002). Variables were log‐transformed and analyzed with the package *caper* (Orme et al., 2013) in the R environment (R_Development_Core_Team 2015). The advantage of using *caper* is that it allows the calculation of the amount of variation in the response variable that is explained by the explanatory variable (*R* ^2^) by comparing the actual model with the intercept‐only model (null model).

As a further test of the robustness of these results, we performed an analysis of the phylogenetic correlation between host diversity and speciosity of genus‐level taxa using MacroCAIC (Agapow & Isaac, 2002). This program extends the method of independent contrasts (Felsenstein, 1985) to generate phylogenetically independent contrasts across the whole phylogeny, investigating whether the species richness of clades correlates with the value of an independent trait. We used the version of MacroCAIC included in *caper*.

As our prediction is a positive correlation between diversity in host use and species numbers in a given clade, we acknowledge a risk of systematic bias in that taxa from poorly described faunas could have few host records as well as few hitherto described species. For this reason, we performed two additional types of analyses.

First, analyses where species numbers only included species found in well‐studied faunas concerning tussock moths, that is, Europe, North America, Japan, and Borneo (and including or excluding three problematic taxa where the species counts in these faunas are consequently uncertain: *Euproctis*,* Olene,* and *Nygmia*). For a conservative test of the robustness of the patterns, we, however, used the entire global host plant range, reasoning that it is the best measure of host use diversity in a given clade. It should be noted that this procedure is conservative in that it could hide a positive correlation (in cases when taxa with high global host diversity are poorly represented in the included faunas) rather than produce it from biased sampling. The collated data can be found in Table S7.

Second, we used the number of host orders used by the single most polyphagous species in the genus‐level taxon (rather than the total number of host orders in the genus) as the predictor of species diversity. This measure should be much less prone to systematic sampling bias.

### 2.6 Related subfamilies of erebidae

In order to investigate whether levels of polyphagy are truly exceptional in Lymantriinae, we contrasted the Lymantriinae with the most closely related subfamilies, according to the results of Zahiri et al. (2012): Hypeninae, Pangraptinae, Herminiinae, Aganainae, and Arctiinae. For these taxa, information was furthermore collected on adult feeding and flight habits of adult females, in an effort to investigate possible causes behind variation in polyphagy (based on earlier suggestions from other groups of moths; Holloway, 1987; Holloway, Barlow, Hok, & Chey, 2013; Janzen, 1984). For those genera included in Zahiri et al. (2012) which could with some confidence be ascribed to one of the aforementioned five subfamilies, host plant records were scrutinized and assembled from the literature and online databases (Chen, 1999; Robinson, Ackery, Kitching, Beccaloni, & Hernández, 2001, 2002). Diversity of host use and frequency of polyphagy were compared between Lymantriinae and the related erebid subfamilies. Furthermore, we include comparisons with three other subfamilies in Erebidae (Calpinae, Scoliopteryginae, and Erebinae), more distant from Lymantriinae, but in some cases also better studied than its closest relatives. For this, we made use of an updated version of an existing dataset from the Oriental region (Robinson, Ackery, Kitching, Beccaloni, & Hernández, 2010; Robinson et al., 2001) showing the number of species in each taxon that feeds on a particular plant family.

### 2.7 Butterflies

We include some comparisons with nymphalid butterflies (using host plant data from Nylin et al. (2014) as this taxon has been the subject of most previous tests of the oscillation hypothesis of diversification. In particular, we show detailed comparisons with the subfamily Nymphalinae, containing several of the most polyphagous butterfly species, in the genera *Vanessa*,* Polygonia,* and *Hypolimnas*.

## 3 RESULTS

### 3.1 General observations from Lymantriinae and butterflies

**Figure 2:**
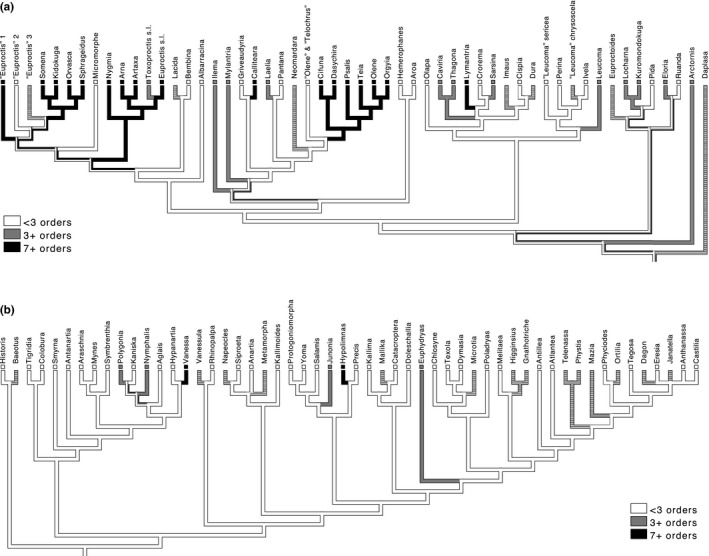
(a) Character optimization of two levels of polyphagy on a phylogeny of the tussock moths subfamily Lymantriinae from (Wang et al. 2015) (b). Character optimization of two levels of polyphagy on a phylogeny of the butterfly subfamily Nymphalinae, based on (Wahlberg, Brower, & Nylin, 2005) and (Nylin & Wahlberg, 2008), with modifications from (Long, Thomson, & Shapiro, 2014). Taxa were coded as having the state 3+ orders if at least one species feed on three orders or more, and 7+ orders if at least one species feed on seven orders or more

Polyphagy at the level of feeding on at least three orders is much more common in Lymantriinae than in butterfly taxa such as Nymphalinae (Figure 2a,b). In the tussock moths even polyphagous species at the level of feeding on at least seven orders are commonly observed, whereas this is very rare in butterflies (Figure 2a,b). In the moths, but not in the butterflies (see also Nylin et al., 2014 for the whole family Nymphalidae), there are entire clades sharing current polyphagy, with character optimization using parsimony suggesting that the patterns reflect ancestral polyphagy that is still retained in related recent taxa.

However, tracing the total number of host orders used by genus‐level clades as a continuous character demonstrates that there is also considerable variation in the level of diversity of hosts used by Lymantriinae moths (Fig. S1; and see Table S2). Several clades have host records from a single plant order, whereas others are known from over thirty host orders. This variation shows that there is scope for the processes suggested by the oscillation hypothesis of diversification to operate in the subfamily and can be used to test the prediction that taxa using more host orders should tend to have more species.

We found Lymantriinae host plant records from 46 different plant orders (42 angiosperm orders, and four rarely used orders outside of the angiosperms), with the most widely used being Malpighiales, Fabales, Fagales, Rosales, and Sapindales (Fig. S2, and Table S1). Contrasting the frequencies of use of different host orders with the same type of data from nymphalid butterflies (Fig. S2) shows how nymphalid host use is strongly dominated by a few host orders. In contrast, host use in tussock moths is much more evenly spread over many host orders, reflecting the overall higher host diversity and polyphagy.

These differences between tussock moths and nymphalid butterflies are reflected also by a much less conservative use of specific host orders in the moths. While the most common host orders for the butterflies are characteristic hosts for entire large clades (Nylin et al., 2014), this is not to the same extent true for the moths. This can be seen by tracing use of the three most common host orders (Malpighiales, Fabales and Fagales, and combinations of these orders) onto the Lymantriinae phylogeny (Fig. S3a) and comparing this with a trace of the most common orders for the subfamily Nymphalinae (Rosales, Lamiales, and Asterales) onto the phylogeny of this clade (Fig. S3b). Whereas the latter analysis shows a clear pattern of ancestral use of Rosales, followed by sequential colonization of Lamiales and later Asterales (Nylin & Wahlberg, 2008), the former shows a much more scattered use of the common hosts, and they are frequently combined.

Furthermore, the phylogenetic signal for polyphagy and for use of specific host orders was very different when comparing tussock moths and nymphalid butterflies (Table 1). In the moths, the phylogenetic signal was very strong for the highest degree of polyphagy studied (feeding on at least seven orders), whereas the host orders had high transition rates, and only a few showed any significant signal. In the butterflies, there was significant phylogenetic signal for the trait “feeding on at least two orders” (typically pairs of taxa commonly and conservatively used together in a given clade) but not for polyphagy as arbitrarily defined here, that is, feeding on at least three orders. In contrast, almost all specific host orders showed very strong and significant signal in the butterflies. This is similar to results in Nylin et al. (2014), where Pagel's lambda was instead used to measure phylogenetic signal.

**Table 1:** Transition rates for polyphagy and for use of specific host orders

Taxon	t.rate	*p*	Taxon	t.rate	*p*
Lymantriinae			Nymphalidae		
3+ orders	0.194	.061	2+ orders	0.0064	**0**
7+ orders	0.076	**0**	3+ orders	0.0034	.066
Malpighiales	0.210	.**049**	Poales	0.0014	**0**
Fabales	0.158	.**007**	Malpighiales	0.0023	**0**
Fagales	0.142	.**006**	Rosales	0.0033	**0**
Rosales	0.299	.093	Solanales	0.0015	**0**
Sapindales	0.273	.084	Lamiales	0.0024	**0**
Myrtales	0.282	.077	Arecales	0.0012	**0**
Ericales	0.154	.**005**	Sapindales	0.0010	**0**
Poales	0.192	.**022**	Laurales	0.0016	.**029**
Malvales	0.247	.064	Ericales	0.0013	.**002**
Lamiales	2.593	.542	Gentianales	0.0005	**0**
Pinales	2.612	.706	Zingiberales	0.0013	.**004**

*p*‐Values show statistical significance of phylogenetic signal (low transition rates) in comparison with simulated data. Significant signal in bold.

### 3.2 Sister‐group comparisons and contrasts in Lymantriinae

Table 2 shows the results of contrasting putative sister clades differing with at least three plant orders in host diversity, with respect to number of species. Of 10 contrasts, nine were positive, and this is a higher number than expected from chance (two‐tailed sign test, *p* < .05). In a paired *t* test (see Methods and Arnqvist et al., 2000; for details), the average of the logarithms of relative species numbers in the contrasts was significantly above zero (average + *SE* = 0.60 + 0.48; *t*_9_ = 3.92, *p* < .01 two‐tailed test). When contrasts were constructed so that even very small differences in host diversity were enough (not shown), the results were not significant in a sign test (8/12 contrasts positive, n.s.) or in a paired *t* test (average ± *SE* = 0.31 + 0.18; *t*_11_ = 1.74, *p* = .11 two‐tailed test). However, we believe that the first set of contrasts (Table 2) are more reliable, as the second contains contrasts such as *Arna* versus *Artaxa* (16 vs. 15 host orders) and *Dasychira* versus *Cifuna* (10 vs. 9 host orders), that is, with differences in host diversity which may easily have arisen from incomplete host records.

**Table 2:** Results of contrasting sister clades differing in host diversity

Sister pairing	HD 1	HD 2	R1	R2	Sign	Rel.	Log
*Somena‐Kidokuga*	22	7	6	2	Pos	3.00	0.477
*(Arna+Artaxa+Toxoproctis+Euproctis)‐Nygmia*	25	15	74	55	Pos	1.35	0.129
*Calliteara‐Griveaudyria*	28	1	45	2	Pos	22.5	1.352
*Laelia‐Pantana*	6	1	100	33	Pos	3.03	0.481
*Orgyia‐Olene*	38	25	62	26	Pos	2.39	0.377
*Aroa‐Hemerophanes*	5	1	18	6	Pos	3.00	0.477
*Lymantria‐(Sarsina+Crorema)*	34	4	170	24	Pos	7.08	0.850
*Leucoma‐(Ivela+Perina+?Leucoma)*	12	7	46	10	Pos	4.60	0.663
*(Locharna+Kuromondokuga)‐Pida*	8	1	9	13	Neg	0.69	−0.160
*Eloria‐Ruanda*	5	1	70	3	Pos	23.3	1.368

HD 1 shows the host diversity of the clade with the highest number of host orders in the contrast, with its corresponding species richness (R1), HD 2 shows the host diversity of the clade with lower number of host orders and its corresponding species richness (R2). Relative richness (Rel.) = (R1/R2) and Log = logarithm of relative richness.

Notably, in each of the ten contrasts in Table 2, the clade with the most diverse host use also contains the most polyphagous species (Table S3, and cf. Table S1). This is not likely to be due to chance (two‐tailed sign test, *p* < .01), but rather suggests that the two measures are correlated in tussock moths. In the other set of contrasts, the same pattern is found (not shown) with the exception of some of the weak contrasts in host diversity such as *Dasychira* versus *Cifuna*. This is perhaps a further indication that these are less reliable, as host diversity (total number of host orders) and maximum polyphagy (highest number of orders in a single species) were overall highly correlated across genus‐level taxa (*r* ^2^ = .904; *p* < .05).

**Figure 3:**
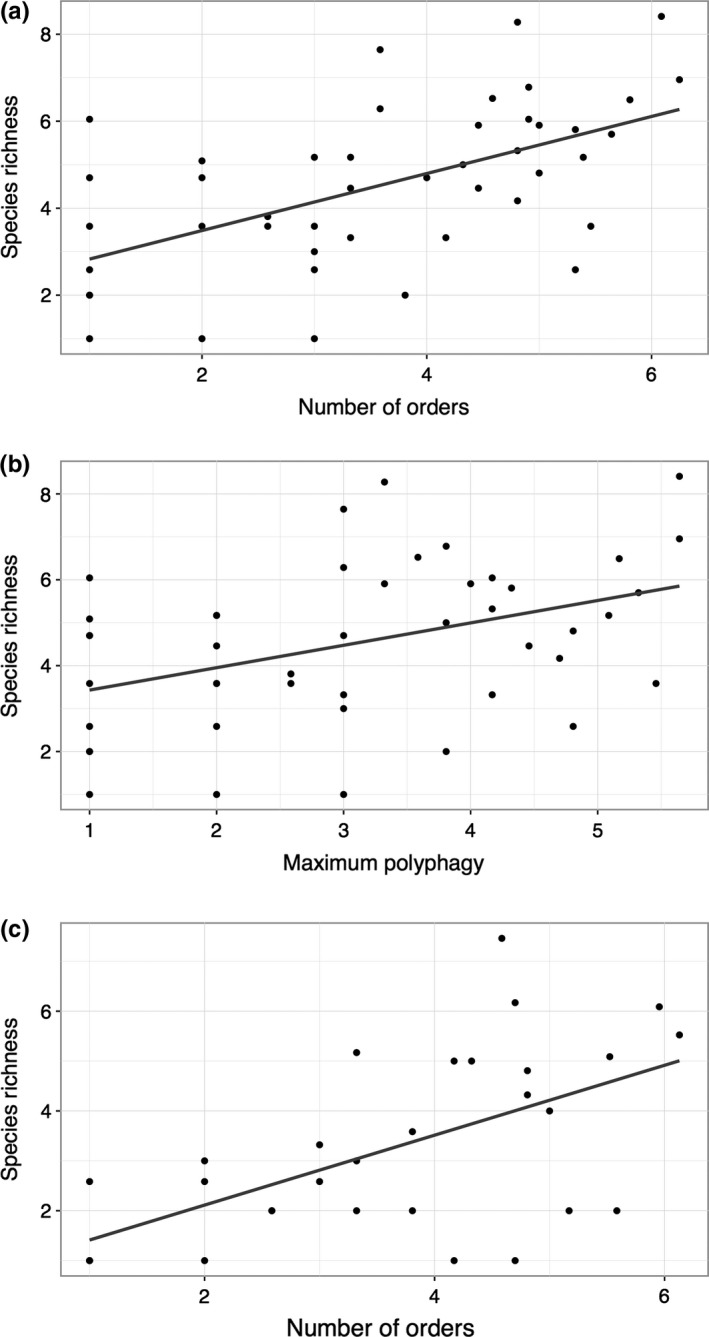
(a) Phylogenetic correlation between host diversity and species richness among genus‐level taxa of Lymantriinae moths. (b) Phylogenetic correlation between maximum species‐level polyphagy and species richness among the same taxa. (c) The same analysis as in (a), but restricted to well‐studied faunas

Furthermore, we found that host diversity has a strong effect on the number of species of Lymantriinae genera (Figure 3a; PGLS: β = 0.77, *SE* = 0.14, *F*_43_ = 30.43, *p* < .001, *R* ^2^ = 0.40) and that phylogeny predicts part of the covariance among trait values (λ = 0.70). Similarly, the MacroCAIC analysis showed a significant correlation between contrasts in host diversity and contrasts in speciosity, respectively (*df *= 42; adjusted *R*‐square = 0.266; *F* = 16.6; *p* < .001).

When the analyses were restricted to the best studied faunas regarding species richness (data set in Table S7), the results were reassuringly similar. The PGLS analysis showed a strong effect of host diversity on species richness (Figure 3b; β = 0.78, *SE* = 0.17, *F*_24_ = 21.35, *p* < .001, *R* ^2^ = 0.45), and the MacroCAIC analysis showed a significant correlation between host diversity and speciosity contrasts (*df* = 24; adjusted *R*‐square = 0.213; *F* = 7.76; *p* < .05). Very similar results were found when the problematic taxa *Euproctis*,* Olene,* and *Nygmia* were included in these analyses (not shown).

Using maximum species‐level polyphagy in a genus‐leve taxon as the predictor of its species richness again gave similar results, in the PGLS analysis (Figure 3c; β = 0.64, *SE* = 0.17, *F*_43_ = 13.87, *p* < .001, *R* ^2^ = 0.23) as well as the MacroCAIC analysis (*df* = 42; adjusted *R*‐square = 0.17; *F* = 9.72; *p* < 0.01).

### 3.3 Comparisons with related subfamilies

Table S4 shows a number of comparisons between host use in Lymantriinae and its closest relatives, exploring host diversity and polyphagy. Note that genera in Lymantriinae tend to have higher average host diversity in terms of total number of host orders, as well as more orders being used by the most polyphagous species in the taxon, but these differences are not significant (*t* tests, *p* = .32 and *p* = .20, respectively). However, genera in Lymantriinae also have a higher average frequency of polyphagous species, both in terms of feeding on at least three, or at least seven orders, and these differences are significant or near‐significant (*t* tests, *p* < .05 and *p* = .05, respectively). This is despite the fact that high frequencies of polyphagy occur also in the related subfamily Arctiinae (Table S4).

Interestingly, polyphagy seems to be closely related to life history, in that having nonfeeding adults is a general characteristic of the Lymantriinae, but also commonly occurs in the Arctiinae (but is not known from other closely related subfamilies; Table S5). Similarly, loss of flight ability in female adults is found in some Lymantriinae and some Arctiinae, but not elsewhere in the clade of related subfamilies, suggesting that it is an evolutionary consequence of the nonfeeding and thus short‐lived adults in these two taxa (Table S5).

Table S6 gives an overview of host use in the oriental tropics in the family Erebidae as a whole (omitting some small subfamilies, as well as Lithosiini of Arctineae because of the unreliable records from these lichen feeders). The data show the number of host genera recorded from each plant family, giving another form of indication of the degree of host specialization. It can be seen that most tribes in Lymantriinae use genera from a diverse set of host families (in line with the analysis above but using a different type of data) and this is true also for parts of Arctiinae—where nonfeeding adults are also common. In contrast, extensively studied taxa with big, mobile, and feeding adults include Calpinae, where host records are dominated by Menispermaceae (Ranunculales); Scoliopteryginae, dominated by Malvaecae (Malvales); and finally Erebinae, where several tribes are strongly dominated by records from Fabaceae (Fabales), whereas other tribes show specialization on other host clades (Table S6). In other words, moth taxa with feeding adults seem to be more butterfly‐like in their host use and life history.

## 4 DISCUSSION

We have shown here that the Lymantriinae stand out in terms of their high levels of polyphagy and diversity of host use, and provide evidence suggesting that this has been a factor in the diversification of this species‐rich taxon, in line with the oscillation hypothesis of host use (Janz & Nylin, 2008) and more generally in line with plasticity as a driver of diversification (Nylin & Janz, 2009; Pfennig et al., 2010; West‐Eberhard, 2003). Due to the difficulties in estimating species numbers when taxonomy is in flux (Zahiri et al., 2012) and the scarcity of fossil Lepidoptera for calibration of dated phylogenies, it is not entirely clear whether Lymantriinae also stands out among relatives in terms of rapid diversification. A dedicated analysis would be necessary to address the question of whether diversification rates are strongly tied to the levels of polyphagy across Lepidoptera.

The degree of polyphagy varies across Lymantriinae, but is overall high compared to its closest relatives, and in particular compared to the nymphalid butterflies which have been the focus of most previous studies testing this particular aspect of the oscillation hypothesis of host use (Hamm & Fordyce, 2015; Janz et al., 2006; Nylin & Wahlberg, 2008; Nylin et al., 2014; Weingartner et al., 2006). A correlate of this difference between tussock moths and butterflies is the much lower level of conservatism—when it comes to utilizing particular host orders over long time spans—that can be observed in the moths. Nevertheless, we have found that the predictions from the oscillation hypothesis of diversification are supported in both taxa.

Comparing the life histories of tussock moths with their relatives in Erebidae (and with butterflies) suggests a likely cause for the ubiquitous polyphagy: the nonfeeding adults in Lymantriinae. Polyphagy seems to be part of a resulting syndrome where adults are short‐lived and females are stationary—sometimes even flightless—and oviposition therefore occurs indiscriminately in masses. This parallels suggestions made earlier by Janzen (1984), Holloway (1987), and Holloway et al. (2013) for another group of moths (the superfamily Bombycoidea), proposing that the general life histories of big moths can be usefully divided into two groups based on whether adults feed or not. The nonfeeding group is also characterized by having mobile males that search for sedentary females, with an associated marked sexual dimorphism. Further, they tend to lay large egg clusters in tree crowns. Moths where both sexes feed, in contrast, are highly active, long‐lived, and show weak or absent sexual dimorphism. They tend to oviposit singly, often on smaller plants, shrubs, or vines. Lymantriinae and some parts of Arctiinae (where levels of polyphagy are also high) belong in the first category, but their other relatives in Erebidae belong in the second. Most butterflies could also be seen as best fitting in this second category, although there is much variation in, for example, clutch size and host plant growth form. Interestingly, butterflies feeding on trees tend to be more diverse in their host use (Janz & Nylin, 1998; testing predictions originating from Feeny, 1976), which suggests that the higher chemical similarity among trees compared to other plant growth forms may be an additional factor facilitating polyphagy in the Lymantriinae and other nonfeeding moths.

We made use of the varying degrees of polyphagy within Lymantriinae to test whether the oscillation hypothesis of diversification can apply even under the very high overall levels of polyphagy, and thus presumably in plasticity, seen in this taxon. As in the previously studied butterflies, we found that clades with higher diversity of hosts contained more species than their putative sister clades. Importantly, the taxa with highest host diversity also consistently contained the most extremely polyphagous single species, in the contrasts between putative sister taxa as well as in the overall dataset. This suggests that diversity of host use in fact reflects actual past and present polyphagy, rather than being an artifact of summing the host use of more specialized species. In other words, this result demonstrates that species‐level polyphagy and host diversity are correlated measures in a taxon where polyphagy is evolutionarily conservative, supporting the use of the latter measure as a proxy for the former when transient polyphagy makes this necessary (e.g., in butterflies, where the correlation between polyphagy and host diversity can only be observed as a tendency: Nylin et al., 2014; Scriber, 2010; Weingartner et al., 2006). Furthermore, it makes the alternative causality unlikely, that is, that clades containing more species will seem to use more host orders simply because they would have a greater probability of being recorded on a diverse array of hosts (Janz et al., 2006). If this was the case, we would have no reason to expect consistently seeing the most polyphagous single species in the more speciose clade.

These findings, together with the significant overall correlations found between host diversity and speciosity (in the full dataset as well as when the analyses were restricted to only well‐studied faunas) and also between maximum species‐level polyphagy and speciosity, suggest that the oscillation hypothesis of diversification can apply even in extreme generalists. In more detail, the hypothesis suggests that diversification is caused by oscillations in host range in either of two ways: via sympatric speciation between host races or via geographic expansion facilitated by polyphagy, followed by local host specialization and speciation which may or may not be further aided by the differences in host use (Janz & Nylin, 2008). Evidence that such processes could occur in Lymantriinae was recently presented from two extremely polyphagous and widespread species. In the brown tail moth *Euproctis chrysorrhoea,* it was found that haplotypes did not in general cluster according to host plants, but host‐associated genotypes—suggesting host races—were observed within locations where populations from different hosts occur in sympatry (Marques, Wang, Svensson, Frago, & Anderbrant, 2014). In the gypsy moth *Lymantria dispar,* Lazarevic et al. (2017) could show that the activity of a range of digestive enzymes differed not only between host plants, but also between populations adapted to one or the other host.

We suggest that such patterns can be seen as windows into a process where developmental plasticity drives diversification, similar to the ideas of West‐Eberhard (2003). Specifically, we believe that (i) in polyphagous species, there is typically plasticity in, for example, enzyme activity enabling the use of different hosts, and also colonization of novel hosts (Celorio‐Mancera et al., 2013, 2016); (2) there is some modularity (sensu West‐Eberhard, 2003) in these plastic responses, so that even if there is much overlap between host responses, there is also a degree of independence, meaning that the “modules” can be fine‐tuned by selection to adapt an insect to better use a particular set of hosts (genetic accommodation sensu West‐Eberhard, 2003), sometimes to the exclusion of others; III) any time some hosts are wholly or even partly excluded by one genetic variant, there is the possibility of some degree of reproductive isolation from other variants that still use them, potentially aiding speciation. As noted above, the oscillation hypothesis of diversification allows for this “host race” route to speciation, but also for other mechanisms with less clear connections to developmental plasticity theory.

The hypothesis under study has also found support from other phytophagous insects, for example, papilionid butterflies (Scriber, 2010), and aphids (Liu, Chen, Huang, Jiang, & Qiao, 2015), as well as from parasite‐host systems in general (Agosta, Janz, & Brooks, 2010). However, two recent studies have examined the data from butterflies in new ways and challenged the support of the hypothesis (Hamm & Fordyce, 2015; Hardy & Otto, 2014). In our opinion, however, these studies show interesting patterns but do not truly test or disprove the oscillation hypothesis (see also Janz et al., 2016). Hardy and Otto (2014) suggested the alternative “musical chairs hypothesis,” predicting that lineages with more “labile” host associations should diversify more rapidly. This is, however, not necessarily in opposition to the oscillation hypothesis. It seems to us that “labile host associations” could in many cases be seen as just another word for phenotypic plasticity allowing for niche shifts (Hoang, Matzkin, & Bono, 2015; Nylin & Janz, 2009), as it is hard to envisage a shift from one host to another without at least some potential to feed on both, and an intermediate stage when both are used. We further believe that such use of more than one host is typically not possible without some phenotypic plasticity to help cope with the varying diets, although the role of plasticity would be less important when resources are very similar across distantly related hosts. Similarly, Hamm and Fordyce (2015) set out to test the prediction that lineages with higher diversification rates should have higher host breadth, and challenged the oscillation hypothesis when not finding it. However, this result is in fact entirely in line with the original publication inspiring the oscillation hypothesis (Janz et al., 2006) where it was shown that although differences in host diversity between sister groups consistently predicts differences in species richness, the opposite is not true—because host use is not necessarily the only or even most important driver of diversification in a given clade.

All attempts at testing the oscillation hypothesis directly are plagued with the severe problems associated with accurately reconstructing host breadth at internal nodes in the phylogeny (Stireman, 2005). The two studies just mentioned tried to avoid these problems by instead testing models of trait‐dependent diversification (BiSSE and related models). This general methodology has recently been severely criticized (Maddison & FitzJohn, 2015; Rabosky & Goldberg, 2015). For this reason, we find sister‐group comparisons and correlations of species richness in clades differing in host diversity to be the most robust and transparent test of the hypothesis that oscillations in host breadth can elevate diversification rates.

## 5 CONCLUSIONS

We have found that predictions from the oscillation hypothesis of a positive relationship between host diversity and diversification are upheld also in Lymantriinae, with its very high overall levels of polyphagy, indicating that it may apply across phytophagous insects and other host–parasite systems. More generally, our results also indicate that even high levels of plasticity can drive diversification, at least when there are oscillations in these levels over evolutionary time.

## ACKNOWLEDGMENTS

This work was supported by Oversea Study Program of Guangzhou Elite Project (Sui jiaoke [2012] 59) to HW, by the Swedish Research Council (2011‐5636 and 2015‐4218) to SN and by the Academy of Finland to NW. Discussions at the symposium “Changing species associations in a changing world: a Marcus Wallenberg symposium” (MWS 2015.0009 to SN) improved the manuscript. We thank Alejandro Gonzalez Voyer for advice on data analysis.

## CONFLICT OF INTEREST

None declared.

## AUTHORS ‘CONTRIBUTIONS

H.W., N.J., M.W., J.D.H., and S.N. designed the study. H.W. collected most of the data; J.D.H. collected additional host and life history data and N.W. collected the phylogenetic data. S.N., H.W., and M.P.B. analyzed the data. H.W. and S.N. drafted the manuscript. All authors contributed to revisions of the manuscript and approved the final version.

## DATA ARCHIVING

All primary data are included in the manuscript or in the supporting information.
