# Supplementary material for: Polyphagy and diversification in tussock moths: Support for the oscillation hypothesis from extreme generalists
Source: Ecol Evol. 2017 Aug 30;7(19):7975–86. doi: 10.1002/ece3.3350 (PMC5632610; doi:10.1002/ece3.3350)
Supplement: Supplementary file 1 [file ECE3-7-7975-s001.pdf]

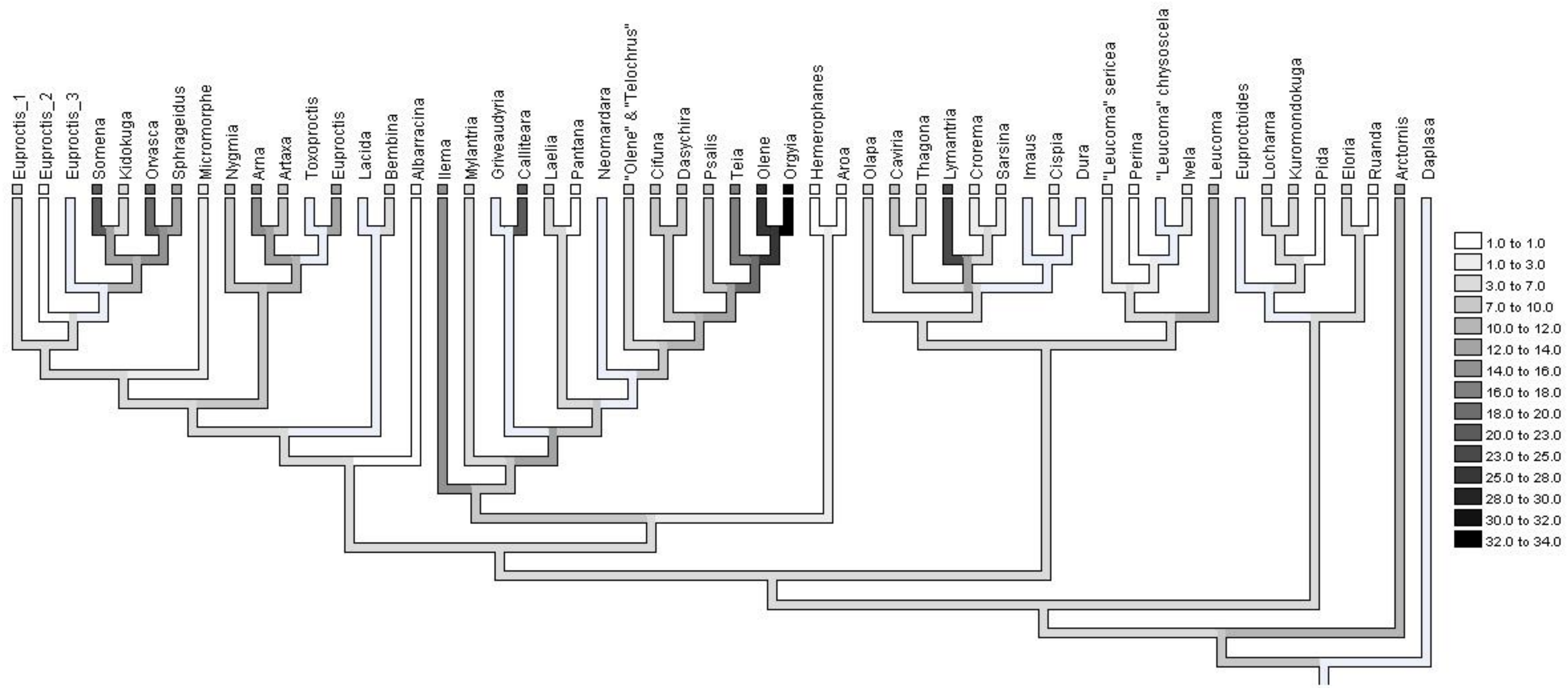

Fig. S1. Character optimization of the total numbers of host plant orders on a phylogeny of the tussock moths subfamily Lymantriinae [25].

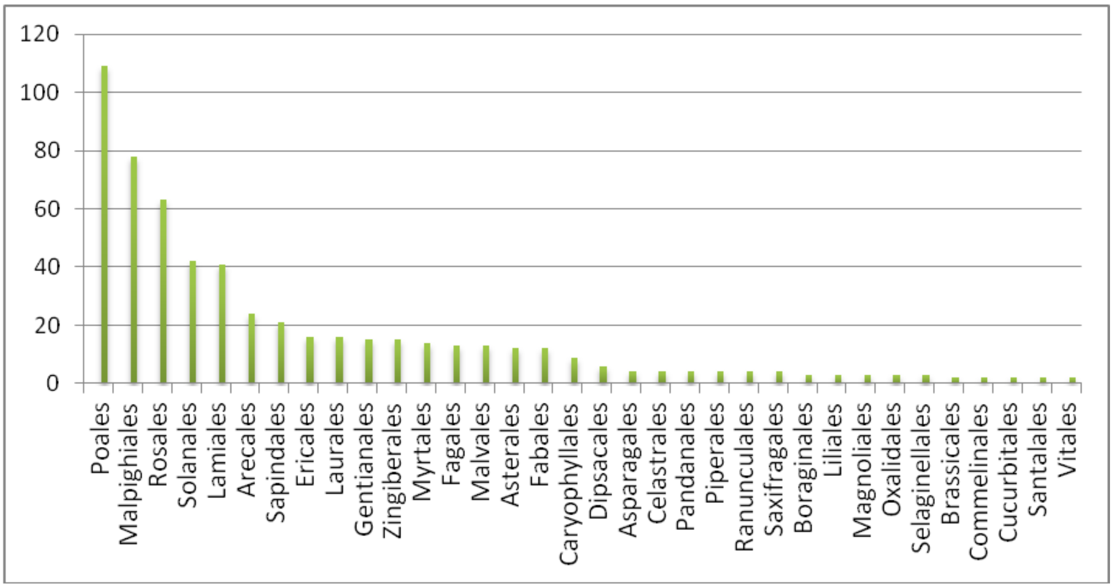

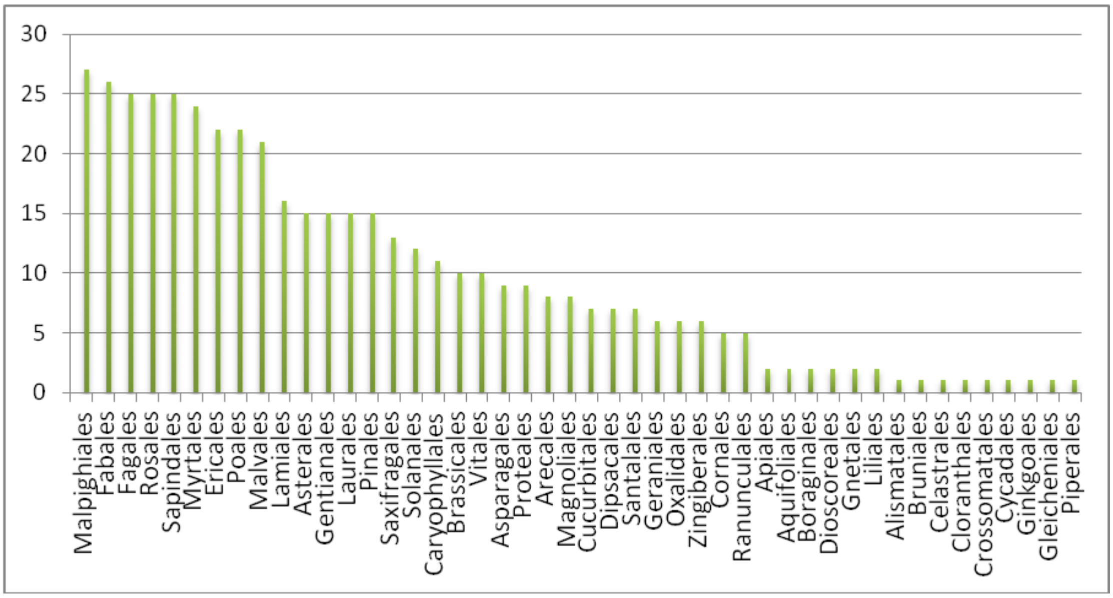

Fig. S2. Frequencies of use of different host orders by genus-level taxa in nymphalid butterflies (top figure, data from [8]) and in tussock moths (bottom figure). Note the different scales of the y-axes.

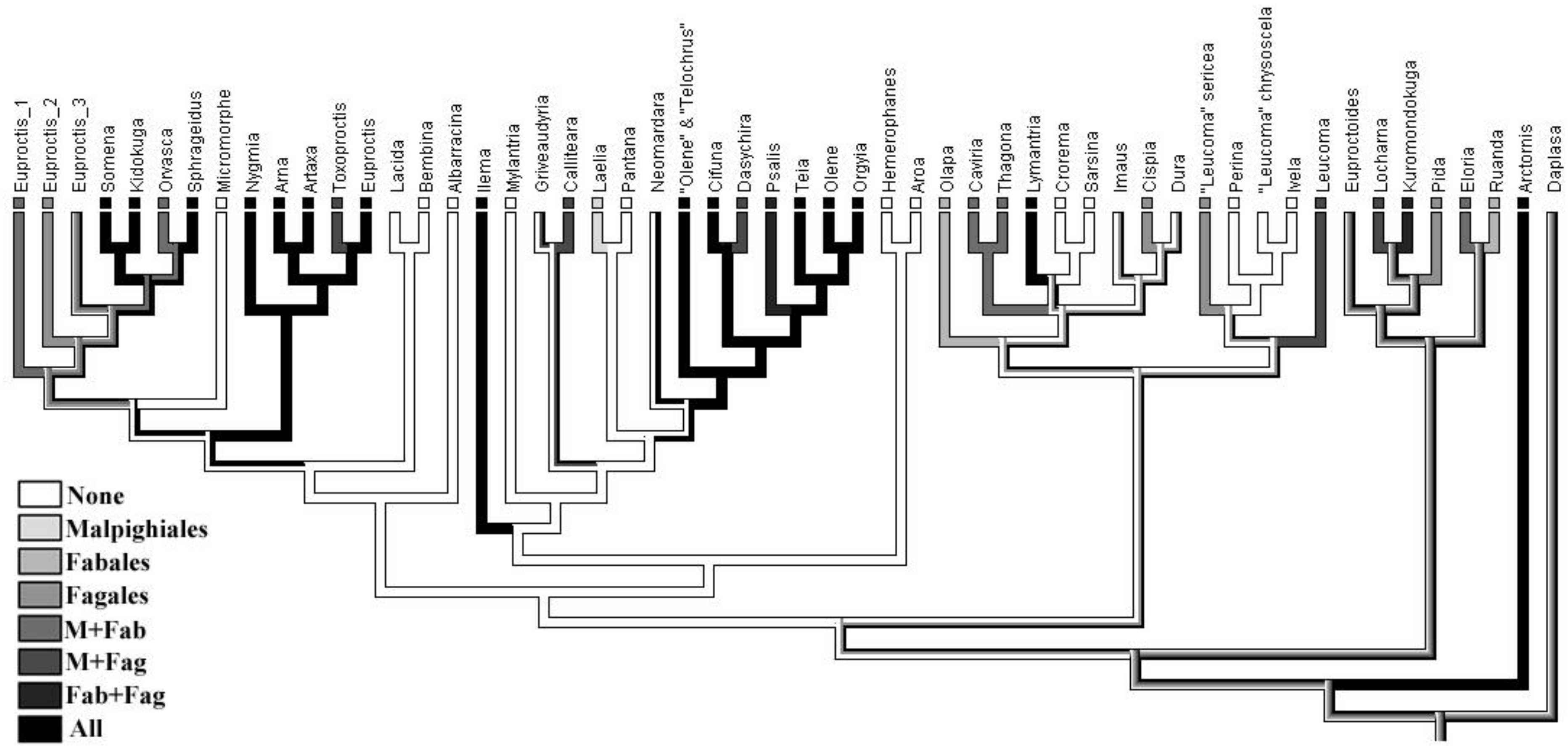

Fig. S3a. Character optimization of use of the three most common host orders, and combinations between them, by the tussock moths subfamily Lymantriinae. Phylogeny from [25].

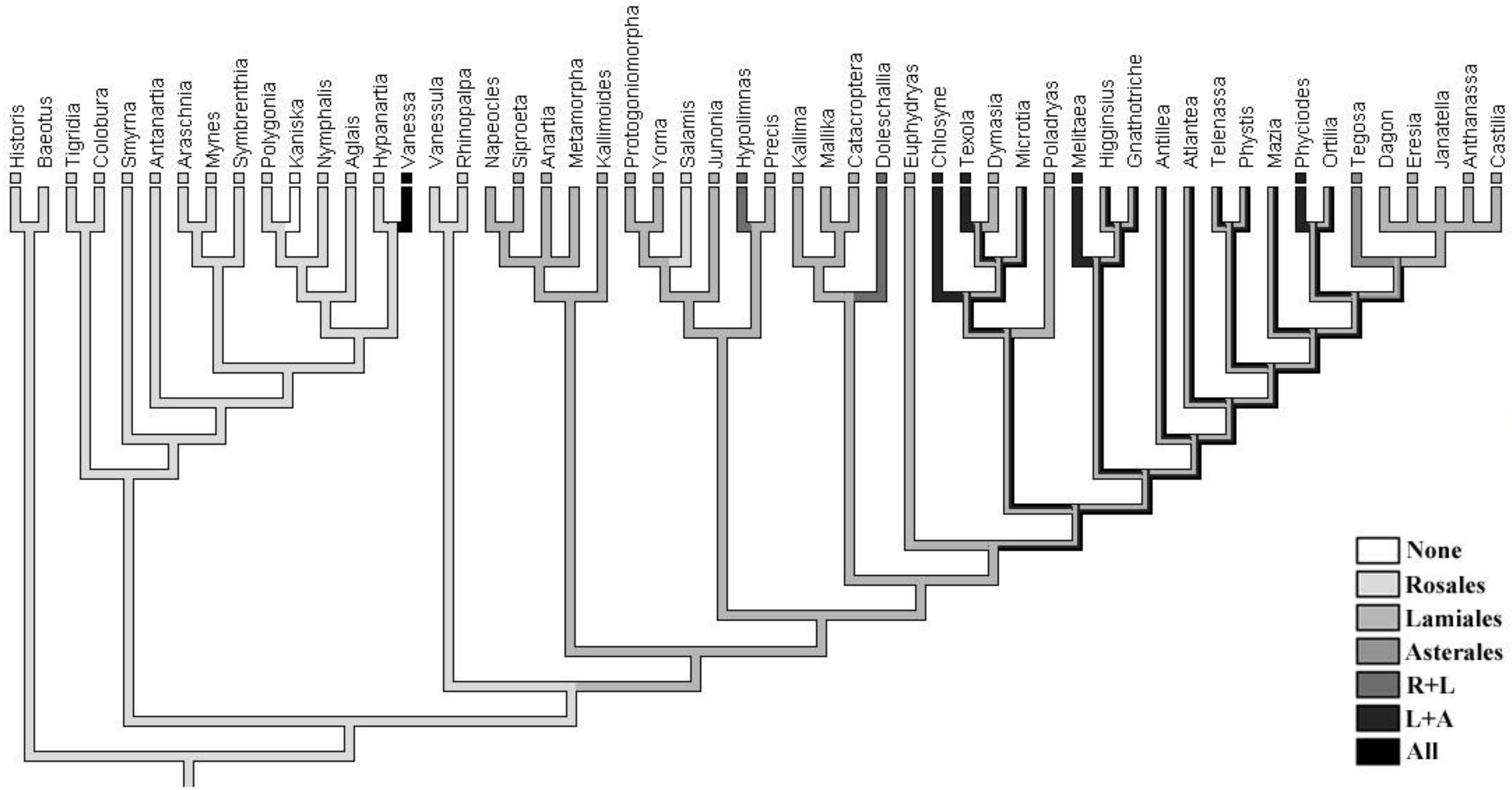

Fig. S3b. Character optimization of use of the three most common host orders, and combinations between them, by the butterfly subfamily Nymphalinae.

Phylogeny from [63] with modifications from [64]. Host data from [10].
