# Supplementary material for: Polyphagy and diversification in tussock moths: Support for the oscillation hypothesis from extreme generalists
Source: Ecol Evol. 2017 Aug 30;7(19):7975–86. doi: 10.1002/ece3.3350 (PMC5632610; doi:10.1002/ece3.3350)
Supplement: Supplementary file 2 [file ECE3-7-7975-s002.pdf]

Table S1. Host plant data at the species levels of Lymantriinae

Lymantriinae taxa	Hostplants (Families)	Hostplants (Orders)
*Daplasa blacklinea**	Missing	Missing
*Daplasa irrorata**	Missing	Missing
Arctornis diaphora *	Missing	Missing
*Arctornis ecnomoda**	Bombacaceae	Malvales
*Arctornis denudata**	Missing	Missing
*Arctornis jonasi**	Fagaceae, Adoxaceae, Rosaceae, Chloranthaceae	Dipsacales, Rosales, Fagales, Chloranthales
*Arctornis discirufa**	Missing	Missing
*Arctornis gracilis**	Missing	Missing
*Arctornis phrika**	Dipterocarpaceae	Malvales
*Arctornis rutila**	Sterculiaceae, Ebenaceae	Ericales, Malvales
*Arctornis l-nigrum**	Fagaceae, Rosaceae, Betulaceae, Salicaceae, Ulmaceae, Tiliaceae	Rosales, Fagales, Malpighiales, Malvales
*Arctornis nivea**	Missing	Missing
*Arctornis submarginata**	Lauraceae,Theaceae, Lythraceae, Combretaceae, Sterculiaceae, Poaceae	Laurales, Ericales, Myrtales, Malvales, Poales
Arctornis subvitrea	Theaceae	Ericales
Arctornis phaeocraspeda	Theaceae	Ericales

Arctornis anserella	Theaceae, Fagaceae	Ericales, Fagales
Arctornis chichibense	Fagaceae	Ericales
Arctornis crocophala	Theaceae	Ericales
Arctornis crgnopsis	Theaceae	Ericales
Arctornis cygna	Theaceae	Ericales
Arctornis diaphana	Dipterocarpaceae	Malvales
Arctornis alba	Theaceae, Fagaceae, Betulaceae	Ericales, Fagales,
Arctornis discipuncta	Combretaceae	Myrtales
Arctornis singaporensis	Dipterocarpaceae, Melastomataceae	Malvales
Arctornis egerina	Euphorbiaceae	Malpighiales
Arctornis galene	Lauraceae	Laurales
Arctornis flavescens	Dipterocarpaceae	Malvales
Arctornis riguata	Anacardiaceae	Sapindales
Arctornis perfecta	Lauraceae, Sapindaceae	Laurales, Sapindales
Arctornis flora	Bombacaceae	Malvales
Arctornis egens?	Elaeocarpaceae	Oxalidales
Arctornis melanocraspis	Rhizophoraceae	Malpighiales

Arctornis marginalis	Anacardiaceae, Combretaceae	Sapindales, Myrtales
Arctornis marginata	Ebenaceae	Ericales
Arctornis spp	Fabaceae, Brassicaceae	Fabales, Brassical es
*Ruanda aetheria**	Missing	Missing
Ruanda nuda	Fabaceae	Fabales
*Eloria onaba**	Erythroxylaceae	Malpighiales
*Eloria torrida**	Erythroxylaceae, Lauraceae, Myrtaceae, Rhizophoraceae	Malpighiales, Laurales, Myrtales
Eloria sp	Fabaceae	Fabales
Eloria captiosa	Erythroxylaceae	Malpighiales
Eloria subapicalis	Erythroxylaceae	Malpighiales
Eloria moeonia	Erythroxylaceae, Fabaceae	Malpighiales, Fabales
Eloria noyesi	Erythroxylaceae	Malpighiales
Eloria spectra	Erythroxylaceae, Fabaceae, Malvaceae	Malpighiales, Fabales, Malvales
*Euproctoides acrisia**	Missing	Missing
*Locharna strigipennis**	Fagaceae, Lauraceae, Anacardiaceae, Clusiaceae	Fagales, Laurales, Sapindales, Malpighiales
Locharna limbata	Leeaceae	Vitales
*Kuromondokuga niphonis**	Fagaceae, Betulaceae, Grossulariaceae, Fabaceae, Rosaceae	Fagales, Saxifragales, Fabales, Rosales

Kuromondokuga albofascia *	Fagaceae, Betulaceae	Fagales
Kuromondokuga disparilis	Betulaceae	Fagales
Kuromondokuga separata *	Missing	Missing
*Pida patrana**	Missing	Missing
*Pida calligramma**	Missing	Missing
*Pida pilodes**	Missing	Missing
*Pida apicalis**	Missing	Missing
Pida spp	Fagaceae	Fagales
*Pida minensis**	Missing	Missing
*Leucoma costalis**	Missing	Missing
*Leucoma salicis**	Salicaceae, Poaceae, Betulaceae, Aceraceae, Cupressaceae, Rosaceae	Malpighiales, Poales, Fagales, Sapindales, Pinales, Rosales
Leucoma ochripes	Euphorbiaceae, Lauraceae, Ulmaceae	Malpighiales, Laurales, Rosales
Leucoma candida	Salicaceae	Malpighiales
Leucoma sartus	Salicaceae	Malpighiales
Leucoma wiltshirei	Fagaceae	Fagales
Leucoma cygna	Lauraceae, Theaceae, Bombacaceae, Magnoliaceae,Sapindaceae	Laurales, Ericales, Malvales, Magnoliales,

		Sapindales
Leucoma ochropoda	Ulmaceae	Rosales
Leucoma parva	Asteraceae	Asterales
*Leucoma sericea**	Juglandaceae,Lauraceae	Fagales, Laurales
*Perina nuda**	Moraceae, Acanthaceae, Anacardiaceae	Rosales, Lamiales, Sapindales
Perina munda	Moraceae	Rosales
*Leucoma chrysoscela**	Missing	Missing
*Ivela auripes**	Cornaceae, Hamamelidaceae	Cornales, Saxifragales
Ivela ochropoda	Ulmaceae	Rosales
Ivela eshanensis	Lauraceae	Laurales
*Olapa ?tatavensis**	Verbenaceae, Bombacaceae,Sterculiaceae	Lamiales, Malvales
Olapa_fulviceps	Malvaceae, Fabaceae	Malvales, Fabales
Olapa nigribasis	Sterculiaceae, Malvaceae	Malvales
Olapa crocicollis	Myrtaceae	Myrtales
Olapa nuda	Fabaceae	Fabales
*Caviria regina**	Arecaceae, Fabaceae, Hypericaceae, Melastomataceae, Myrtaceae; Salicaceae, Vochysiaceae	Arecales, Fabales, Malpighiales, Myrtales

Caviria spp	Malpighiaceae, Caryocaraceae, Connaraceae, Ebenaceae, Erythroxylaceae, Asteraceae, Myrsinaceae, Proteaceae	Malpighiales, Oxalidales, Ericales, Arecales, Proteales
*Thagona errans**	Myrtaceae	Myrtales
*Thagona tibialis**	Euphorbiaceae, Combretaceae, Fabaceae, Melastomataceae, Sapindaceae, Vochysiaceae	Malpighiales, Myrtales, Fabales, Sapindales
Thagona roseidorsum	Rosaceae	Rosales
Thagona parthenica	Bignoniaceae, Anacardiaceae	Lamiales, Sapindales
*Imaus munda**	Missing	Missing
*Cispia lunata**	Fagaceae	Fagales
Cispia alba	Meliaceae	Sapindales
Cispia charma	Meliaceae	Sapindales
Cispia puncticilia	Sapindaceae	Sapindales
*Dura ippoline**	Missing	Missing
*Crorema ?jordani**	Missing	Missing
Crorema fuscinotata	Poaceae	Poales
Crorema mentiens	Combretaceae	Myrtales
*Sarsina purpurascens**	Myrtaceae	Myrtales

Sarsina festiva	Myrtaceae	Myrtales
Sarsina violascens	Myrtaceae, Asteraceae, Oleaceae	Myrtales, Asterales, Lamiales
*Lymantria fergusoni**	Missing	Missing
*Lymantria furvinis**	Missing	Missing
*Lymantria nigriplagiata**	Missing	Missing
*Lymantria grisea**	Missing	Missing
*Lymantria tortivalvula**	Missing	Missing
*Lymantria mathura**	Rubiaceae, Fagaceae, Lythraceae, Hamamelidaceae, Anacardiaceae, Lecythidaceae, Dipterocarpaceae, Myrtaceae, Combretaceae, Ulmaceae, Rosaceae, Euphorbiaceae	Gentianales, Fagales, Myrtales, Saxifragales, Sapindales, Ericales, Malvales, Rosales, Malpighiales
*Lymantria beatrix**	Lythraceae, Anacardiaceae, Euphorbiaceae, Punicaceae, Bombacaceae	Myrtales, Sapindales, Malpighiales, Malvales
*Lymantria hauensteini**	Missing	Missing
*Lymantria dispar**	Araucariaceae, Cupressaceae, Pinaceae, Taxaceae, Taxodiaceae, Aceraceae, Betulaceae, Rosaceae, Araliaceae, Berberidaceae, Corylaceae, Juglandaceae, Fagaceae, Bignoniaceae, Ulmaceae, Asteraceae, Rutaceae, Clethraceae, Myricaceae, Cornaceae, Caprifoliaceae, Ebenaceae, Oleaceae, Ericaceae, Ginkgoaceae, Fabaceae,	Pinales, Sapindales, Fagales, Rosales, Apiales, Ranunculales, Lamiales, Asterales, Ericales, Cornales, Dipsacales, Ginkgoales, Fabales, Saxifragales, Aquifoliales, Asparagales, Laurales, Magnoliales, Cornales, Proteales,

	Hamamelidaceae, Aquifoliaceae, Iridaceae, Lauraceae, Moraceae, Magnoliaceae, Cornaceae, Anacardiaceae, Platanaceae, Salicaceae, Grossulariaceae, Polygonaceae, Tamaricaceae, Araceae, Tiliaceae, Vitaceae, Poaceae, Rhamnaceae	Malpighiales, Saxifragales, Caryophyllales, Alismatales, Malvales, Vitales, Poales
*Lymantria schaeferi**	Missing	Missing
*Lymantria concolor**	Fagaceae, Rosaceae	Fagales, Rosales
*Lymantria dissoluta**	Pinaceae, Cupressaceae, Fagaceae	Pinales, Fagales
*Lymantria monacha**	Combretaceae, Dipterocarpaceae, Salicaceae, Fagaceae, Pinaceae, Oleaceae, Betulaceae, Rosaceae, Ulmaceae, Aceraceae, Tiliaceae	Myrtales, Malvales, Malpighiales, Fagales, Pinales, Lamiales, Rosales, Sapindales
*Lymantria similis**	Missing	Missing
Lymantria lepcha	Dipterocarpaceae, Sonneratiaceae, Lythraceae, Combretaceae	Malvales, Myrtales
Lymantria brunneiplaga	Combretaceae, Asteraceae, Convolvulaceae, Cyperaceae, Clusiaceae, Piperaceae, Ulmaceae, Fabaceae, Meliaceae, Euphorbiaceae, Sapotaceae, Turneraceae, Mimosaceae	Myrtales, Asterales, Solanales, Poales, Malpighiales, Piperales, Rosales, Fabales, Sapindales, Ericales
Lymantria ganara	Myrtaceae, Fabaceae, Casuarinaceae, Euphorbiaceae, Rubiaceae, Meliaceae	Myrtales, Fabales, Fagales, Malpighiales, Gentianales, Sapindales
Lymantria incerta	Oleaceae, Sapindaceae, Fabaceae, Moraceae, Combretaceae,	Lamiales, Sapindales, Fabales, Rosales,

	Rhamnaceae	Myrtales
Lymantria singapura	Pinaceae	Pinales
Lymantria fumida	Cupressaceae, Pinaceae	Pinales
Lymantria umbrifera	Hamamelidaceae	Saxifragales
Lymantria minora	Anacardiaceae, Euphorbiaceae, Fagaceae, Hamamelidaceae, Rosaceae, Ulmaceae, Combretaceae, Dipterocarpaceae, Rubiaceae, Myrtaceae, Fabaceae	Sapindales, Malpighiales, Fagales, Saxifragales, Rosales, Myrtales, Malvales, Genitanales, Fabales
Lymantria juglandis	Juglandaceae	Fagales
Lymantria minomonis	Cupressaceae, Pinaceae, Fagaceae	Pinales, Fagales
Lymantria serva	Moraceae, Combretaceae, Dipterocarpaceae, Lythraceae, Myrtaceae, Verbenaceae	Rosales, Myrtales, Malvales, Lamiales
Lymantria viola	Oleaceae, Fagaceae, Rosaceae, Combretaceae, Burseraceae, Rubiaceae	Lamiales, Fagales, Rosales, Myrtales, Sapindales, Gentianales
Lymantria orestera	Alangiaceae	Cornales
Lymantria nebulosa	Aceraceae, Hamamelidaceae	Sapindales, Saxifragales
Lymantria servula	Pinaceae	Pinales
Lymantria marginalis	Myrtaceae, Anacardiaceae, Dipterocarpaceae	Myrtales, Sapindales, Malvales

Lymantria marginata	Anacardiaceae, Myrtaceae, Dipterocarpaceae, Bombacaceae	Sapindales, Myrtales, Malvales
Lymantria bantaizana	Juglandaceae	Fagales
Lymantria lucescens	Fagaceae	Fagales
Lymantria umbrosa	Fabaceae	Fabales
Lymantria pelospila	Myrtaceae	Myrtales
Lymantria ascetria	Fabaceae, Rosaceae	Fabales, Rosales
Lymantria ampla	Arecaceae, Rubiaceae, Anacardiaceae, Begoniaceae, Fabaceae, Apocynaceae, Casuarinaceae, Myrtaceae, Moraceae, Malvaceae, Lythraceae, Rosaceae, Geraniaceae, Combretaceae, Euphorbiaceae, Dipterocarpaceae, Verbenaceae, Sterculiaceae	Arecales, Gentianales, Sapindales, Cucurbitales, Fabales, Fagales, Myrtales, Rosales, Malvales, Geraniales, Malpighiales, Lamiales
Lymantria destituta	Rosaceae, Casuarinaceae	Rosales, Fagales
Lymantria atemeles	Anacardiaceae, Arecaceae	Sapindales, Arecales
Lymantria bivittata	Lythraceae, Lecythidaceae, Fagaceae, Dipterocarpaceae, Combretaceae	Myrtales, Ericales, Fagales, Malvales
Lymantria grandis	Moraceae, Lecythidaceae, Dipterocarpaceae	Rosales, Ericales, Malvales
Lymantria lunata	Anacardiaceae, Rutaceae, Ebenaceae, Myrtaceae, Moraceae, Euphorbiaceae, Fabaceae, Combretaceae	Sapindales, Ericales, Myrtales, Rosales, Malpighiales, Fabales
Lymantria loacana	Pinaceae	Pinales

Lymantria lapidicola	Pinaceae, Rosaceae	Pinales, Rosales
Lymantria modesta	Capparaceae, Anacardiaceae	Brassicales, Sapindales
Lymantria nigra	Loranthaceae, Anacardiaceae	Santalales, Sapindales
Lymantria ninayi	Pinaceae	Pinales
Lymantria novaguinensis	Pinaceae	Pinales
Lymantria rosina	Myrtaceae	Myrtales
Lymantria nebulosa	Hamamelidaceae	Saxifragales
Lymantria obfuscata	Betulaceae, Rosaceae, Juglandaceae, Salicaceae, Fagaceae, Sterculiaceae	Fagales, Rosales, Malpighiales, Malvales
Lymantria singapura	Pinaceae	Pinales
Lymantria sobrina	Pinaceae	Pinales
Lymantria sphalera	Combretaceae	Myrtales
Lymantria subrosea	Dipterocarpaceae	Malvales
Lymantria todara	Lythraceae, Dipterocarpaceae, Combretaceae	Myrtales, Malvales
Lymantria viola	Combretaceae, Rubiaceae	Malvales, Gentianales
Lymantria detersa	Casuarinaceae	Fagales
Lymantria obliqualinea	Cannaceae	Zingiberales
Lymantria semicincta	Dipterocarpaceae	Malvales

Lymantria xylina	Casuarinaceae, Fabaceae, Simaroubaceae, Fagaceae, Juglandaceae, Salicaceae, Myrtaceae, Malvaceae, Meliaceae, Altingiaceae, Euphorbiaceae, Paulowniaceae, Sapindaceae, Rosaceae, Moraceae, Lythraceae, Ebenaceae,Theaceae, Hamamelidaceae, Mimosaceae, Sterculiaceae, Scrophulariaceae, Punicaceae	Fagales, Fabales, Sapindales, Malpighiales, Myrtales, Malvales, Saxifragales, Lamiales, Rosales, Ericales
*Aroa discalis**	Poaceae	Poales
Aroa danva	Asteraceae	Asterales
Aroa difficilis	Combretaceae, Ebenaceae	Myrtales, Ericales
Aroa interrogationis	Poaceae	Poales
Aroa melanoleuca	Myrtaceae, Pinaceae	Myrtales, Pinales
*Hemerophanes diatoma**	Missing	Missing
Hemerophanes enos	Combretaceae	Myrtales
Hemerophanes libyra	Combretaceae	Myrtales
*Ilema anaha**	Missing	Missing
*Ilema costalis**	Missing	Missing
*Ilema catocaloides**	Missing	Missing
*Ilema jankowskii**	Vitaceae, Rosaceae, Grossulariaceae	Vitales, Rosales, Saxifragales

Ilema vaneeckei	Rutaceae, Rubiaceae	Sapindales, Gentianales
Ilema baruna	Fabaceae, Dipterocarpaceae, Melastomataceae, Rubiaceae	Fabales, Malvales, Myrtales, Gentianales
Ilema preangerensis	Orchidaceae	Asparagales
Ilema inclusa	Malvaceae, Loganiaceae, Melastomataceae, Lauraceae	Malvales, Gentianales, Myrtales, Laurales
Ilema sp	Gleicheniaceae	Gleicheniales
Ilema chalana	Fabaceae	Fabales
Ilema nachiensis	Lauraceae, Fagaceae, Betulaceae, Cornaceae, Euphorbiaceae, Boraginaceae, Malvaceae, Loganiaceae, Melastomataceae	Laurales, Fagales, Cornales, Malpighiales, Boraginales, Malvales, Gentianales, Myrtales
Ilema eurydice	Vitaceae, Rosaceae, Hydrangeaceae, Actinidiaceae, Clethraceae	Vitales, Rosales, Cornales, Ericales
*Mylantria xanthospila**	Rubiaceae, Pinaceae,Asteraceae, Poaceae	Gentianales, Pinales, Asterales, Poales
*Griveaudyria ?cangia**	Missing	Missing
Griveaudyria ila	Mimosaceae	Fabales
*Calliteara grotei**	Fabaceae, Berberidaceae, Myrtaceae, Lythraceae, Euphorbiaceae, Rosaceae, Lauraceae, Polygonaceae, Salicaceae, Fagaceae, Salicaceae, Verbenaceae, Dipterocarpaceae, Combretaceae, Malvaceae	Fabales, Ranunculales, Myrtales, Malpighiales, Rosales, Laurales, Caryophyllales, Fagales, Malvales, Lamiales
*Calliteara melli**	Pinaceae, Cupressaceae	Pinales
*Calliteara contexta**	Missing	Missing

*Calliteara angulata**	Fagaceae	Fagales
*Calliteara complicata**	Missing	Missing
*Calliteara taiwana**	Fagaceae	Fagales
*Calliteara wolongensis**	Missing	Missing
Calliteara argentata	Taxodiaceae, Cupressaceae	Pinales
Calliteara Kikuchii	Fagaceae	Fagales
Calliteara lunulata	Fagaceae	Fagales
Calliteara farenoides	Combretaceae	Myrtales
Calliteara pseudabietis	Rosaceae, Salicaceae, Fagaceae, Aceraceae, Juglandaceae, Cornaceae, Caprifoliaceae, Ebenaceae, Lauraceae, Polygonaceae, Pinaceae, Cupressaceae	Rosales, Malpighiales, Fagales, Sapindales, Cornales, Dipsacales, Ericales, Laurales, Caryophyllales, Pinales
Calliteara baibarana	Theaceae	Ericales
Calliteara abietis	Pinaceae, Cupressaceae, Taxodiaceae	Pinales
Calliteara pura	Myrtaceae, Pinaceae, Rosaceae, Amaryllidaceae, Musaceae, Magnoliaceae	Myrtales, Pinales, Rosales, Zingiberales, Magnoliales, Asparagales
Calliteara axutha	Pinaceae	Pinales

Calliteara horsfieldi	Paulowniaceae, Salicaceae, Platanaceae, Ulmaceae, Fabaceae, Lauraceae, Euphorbiaceae, Rosaceae, Mimosaceae, Anacardiaceae, Begoniaceae, Bixaceae, Brassicaceae, Theaceae, Arecaceae, Rubiaceae, Myrtaceae, Lythraceae, Verbenaceae, Sapotaceae, Clusiaceae, Tiliaceae, Musaceae, Ericaceae, Sapindaceae, Combretaceae, Sterculiaceae, Hamamelidaceae, Malvaceae, Casuarinaceae, Dipterocarpaceae, Moraceae, Pinaceae, Sapotaceae	Lamiales, Malpighiales, Proteales, Rosales, Fabales, Laurales, Sapindales, Cucurbitales, Malvales, Brassicales, Ericales, Arecales, Gentianales, Myrtales, Zingiberales, Saxifragales, Fagales, Pinales,
Calliteara queenslandica	Pinaceae	Pinales,
Calliteara strigata	Fagaceae	Fagales
Calliteara thwaitesii	Anacardiaceae, Theaceae, Fabaceae, Lecythidaceae, Dipterocarpaceae	Sapindales, Ericales, Fabales, Malvales
Calliteara varia	Lamiaceae	Lamiales
Calliteara fidjiensis	Myrtaceae, Rhizophoraceae	Myrtales, Malpighiales
Calliteara salomonis	Combretaceae	Myrtales
Calliteara cerigoides	Dipterocarpaceae, Myrtaceae	Malvales, Myrtales
Calliteara pudibunda	Fagaceae, Salicaceae, Betulaceae, Rosaceae, Geraniaceae, Aceraceae, Corylaceae, Ericaceae, Cannabaceae, Juglandaceae, Tiliaceae, Ulmaceae, Santalaceae	Fagales, Malpighiales, Rosales, Geraniales, Sapindales, Ericales, Malvales, Santalales

Calliteara conjuncta	Fagaceae,Salicaceae, Euphorbiaceae, Fabaceae, Altingiaceae, Pinaceae, Theaceae	Fagales, Malpighiales, Fabales, Pinales, Ericales, Saxifragales
Calliteara spp	Rutaceae, Bruniaceae, Poaceae	Sapindales, Bruniales, Poales
*Laelia coenosa**	Poaceae, Juncaceae, Cyperaceae, Ulmaceae, Salicaceae, Asteraceae	Poales, Rosales, Malpighiales, Asterales
*Laelia umbrina**	Poaceae	Poales
Laelia suffusa	Poaceae, Cyperaceae	Poales
Laelia gigantea	Poaceae, Cyperaceae	Poales
Laelia devestita	Poaceae	Poales
Laelia extorta	Moraceae	Rosales
Laelia adalia	Poaceae	Poales
Laelia calamaria	Arecaceae	Arecales
Laelia litura	Poaceae	Poales
Laelia subrosea	Poaceae	Poales
Laelia venosa	Arecaceae	Arecales
Laelia fasciata	Poaceae	Poales
Laelia figlina	Cyperaceae, Juncaceae	Poales
Laelia fracta	Poaceae, Cyperaceae, Pinaceae	Poales, Pinales

Laelia flandria	Arecaceae	Arecales
Laelia exclamationis	Poaceae, Cannaceae	Poales, Zingiberales
*Pantana substrigosa**	Poaceae	Poales
*Pantana sinica**	Poaceae	Poales
*Pantana visum**	Poaceae	Poales
Pantana nigrolimbata	Poaceae	Poales
Pantana pluto	Poaceae	Poales
Pantana adara	Poaceae	Poales
Pantana leucogramma	Poaceae	Poales
Pantana ochripalpis	Poaceae	Poales
Pantana simplex	Poaceae	Poales
Pantana phyllostachysae	Poaceae	Poales
*Neomardara africana**	Missing	Missing
*Dasychira glaucozona**	Missing	Missing
*Olene dudgeoni**	Rutaceae, Euphorbiaceae	Sapindales, Malpighiales
Telochurus sp	Fagaceae, Rosaceae, Fabaceae	Fagales, Fabales, Rosales
*Cifuna locuples**	Poaceae, Salicaceae, Rosaceae, Fagaceae, Polygonaceae, Saxifragaceae,	Poales, Malpighiales, Rosales, Fagales, Fabales,

	Vitaceae, Ulmaceae, Fabaceae, Theaceae, Ebenaceae,	Caryophyllales, Saxifragales, Vitales, Ericales
*Dasychira tephra**	Fagaceae	Fagales
Dasychira dorsipennata	Aceraceae, Hippocastanaceae, Rosaceae, Betulaceae, Juglandceae, Fagaceae, Corylaceae, Salicaceae, Ulmaceae	Rosales, Fagales, Malpighiales, Sapindales
Dasychira vagans	Pinaceae, Salicaceae, Aceraceae, Betulaceae, Rosaceae, Fagaceae, Ulmaceae	Rosales, Sapindales, Malpighiales, Fagales, Pinales
Dasychira atrivenosa	Hamamelidaceae	Saxifragales
Dasychira basiflava	Betulaceae, Juglandaceae, Rosaceae, Fagaceae, Tiliaceae, Ulmaceae, Ericaceae	Rosales, Fagales, Malvales, Ericales
Dasychira meridionalis	Juglandaceae, Fagaceae, Euphorbiaceae, Rosaceae, Ulmaceae	Rosales, Fagales, Malpighiales
Dasychira cinnamomea	Fagaceae, Cyperaceae, Juglandaceae, Rosaceae, Corylaceae, Ulmaceae	Rosales, Fagales, Poales
Dasychira leucophaea	Betulaceae, Juglandaceae, Ebenaceae, Hamamelidaceae, Rosaceae, Pinaceae, Fagaceae	Rosales, Fagales, Ericales, Saxifragales, Pinales
Dasychira obliquata	Aceraceae, Betulaceae, Juglandaceae, Fagaceae, Rosaceae, Ulmaceae, Ericaceae	Sapindales, Fagales, Rosales, Ericales
Dasychira dominickaria	Taxodiaceae	Pinales
Dasychira plagiata	Aceraceae, Betulaceae, Asteraceae, Cupressaceae, Pinaceae, Fabaceae,	Sapindales, Fagales, Rosales, Pinales, Asterales,

	Salicaceae, Rosaceae, Fagaceae, Taxodiaceae, Tiliaceae, Ulmaceae	Malpighiales, Malvales
Dasychira pinicola	Pinaceae	Pinales
Dasychira grisefacta	Pinaceae, Asteraceae, Cupressaceae, Taxodiaceae	Pinales, Asterales
Dasychira manto	Pinaceae	Pinales
*Psalis pennatula**	Poaceae, Brassicaceae, Theaceae, Cyperaceae, Cucurbitaceae, Casuarinaceae, Lauraceae, Dipterocarpaceae, Solanaceae, Verbenaceae, Fabaceae, Pinaceae	Poales, Ericales, Brassicales, Cucurbitales, Fagales, Laurales, Malvales, Solanales, Lamiales, Fabales, Pinales
*Teia parallela**	Platanaceae, Euphorbiaceae, Fagaceae	Proteales, Malpighiales, Fagales
Teia anartoides	Mimosaceae, Fabaceae, Tamaricaceae, Asteraceae, Solanaceae, Brassicaceae, Rosaceae, Cupressaceae, Asteraceae, Myrtaceae, Santalaceae, Rubiaceae, Iridaceae, Verbenaceae, Musaceae, Passifloraceae, Pinaceae, Platanaceae, Primulaceae, Salicaceae, Ulmaceae, Geraniaceae, Proteaceae	Fabales, Caryophyllales, Asterales, Solanales, Brassicales, Rosales, Pinales, Myrtales, Santalales, Gentianales, Asparagales, Lamiales, Zingiberales, Malpighiales, Proteales, Ericales, Geraniales
Teia prisca	Elaeagnaceae	Rosales
Teia gonostigma	Rosaceae, Rutaceae, Platanaceae, Salicaceae, Betulaceae, Ulmaceae, Fagaceae, Corylaceae, Paulowniaceae, Pinaceae	Rosales, Malpighiales, Fagales, Proteales, Lamiales, Pinales
Teia athlophora	Mimosaceae, Proteaceae, Fabaceae	Fabales, Proteales

Teia trigotephras	Fabaceae, Fagaceae	Fabales, Fagales
Teia ericae	Ericaceae, Salicaceae, Fagaceae, Rosaceae, Elaeagnaceae, Fabaceae, Myricaceae, Rhamnaceae, Tamaricaceae	Ericales, Fagales, Malpighiales, Rosales, Fabales, Caryophyllales
Teia spp	Sterculiaceae,Chenopodiaceae, Euphorbiaceae, Anacardiaceae, Poaceae	Malpighiales, Malvales, Caryophyllales, Sapindales, Poales
*Olene inclusa**	Annonaceae, Averrhoaceae, Bombacaceae, Dipterocarpaceae, Euphorbiaceae, Leeaceae, Geraniaceae, Fabaceae, Moraceae, Musaceae, Myrtaceae, Rosaceae, Rutaceae, Sterculiaceae, Tiliaceae,Verbenaceae, Oxalidaceae, Casuarinaceae, Lauraceae, Arecaceae, Rubiaceae, Anacardiaceae, Orchidaceae, Solanaceae, Poaceae	Magnoliales, Oxalidales, Malvales, Malpighiales, Vitales, Geraniales, Fabales, Rosales, Zingiberales, Myrtales, Sapindales, Lamiales, Fagales, Laurales, Arecales, Gentianales, Asparagales, Solanales, Poales
Olene mendosa	Anacardiaceae, Bombacaceae, Combretaceae, Brassicaceae, Lythraceae, Dipterocarpaceae, Euphorbiaceae, Geraniaceae, Fabaceae, Malvaceae, Rhamnaceae, Rosaceae, Santalaceae, Sapindaceae, Sapotaceae, Solanaceae, Theaceae, Verbenaceae, Poaceae, Rubiaceae, Sterculiaceae, Salicaceae, Lecythidaceae, Pinaceae, Lauraceae, Musaceae, Moraceae, Moringaceae, Myrtaceae, Tiliaceae, Lamiaceae, Proteaceae, Loranthaceae, Myrsinaceae, Iridaceae, Ebenaceae, Marantaceae,	Sapindales, Malvales, Myrtales, Brassicales, Malpighiales, Geraniales, Fabales, Rosales, Santalales, Ericales, Solanales, Lamiales, Poales, Gentianales, Pinales, Laurales, Zingiberales, Proteales, Asparagales, Arecales

	Flacourtiaceae, Arecaceae, Cannaceae, Apocynaceae, Urticaceae, Alliaceae, Asparagaceae	
*Orgyia antiqua**	Pinaceae, Aceraceae, Betulaceae, Rosaceae, Apiaceae, Asteraceae, Fabaceae, Saxifragaceae, Corylaceae, Cucurbitaceae, Fagaceae, Rhamnaceae, Oleaceae, Geraniaceae, Cannabaceae, Plantaginaceae, Salicaceae, Grossulariaceae, Polygonaceae, Cupressaceae, Tiliaceae, Ulmaceae, Ericaceae, Scrophulariaceae, Adoxaceae, Berberidaceae	Pinales, Sapindales, Fagales, Rosales, Apiales, Asterales, Fabales, Saxifragales, Cucurbitales, Lamiales, Geraniales, Malpighiales, Malvales, Caryophyllales, Ericales, Dipsacales, Ranunculales
*Orgyia povera**	Anacardiaceae, Fabaceae, Malpighiaceae	Sapindales, Fabales, Malpighiales
*Orgyia osseata**	Anacardiaceae, Averrhoaceae, Bombacaceae, Dipterocarpaceae, Fabaceae, Myrtaceae, Rubiaceae, Sapindaceae, Sonneratiaceae, Sterculiaceae, Annonaceae, Euphorbiaceae, Theaceae, Lauraceae, Vitaceae, Melastomataceae, Sapotaceae, Lecythidaceae, Euphorbiaceae, Apocynaceae, Sterculiaceae, Poaceae, Rhamnaceae, Rosaceae	Sapindales, Oxalidales, Malvales, Fabales, Myrtales, Gentianales, Magnoliales, Malpighiales, Ericales, Laurales, Vitales, Poales, Rosales
Orgyia araea	Fabaceae, Ebenaceae, Cannaceae, Rubiaceae, Moraceae	Fabales, Ericales, Zingiberales, Gentianales, Rosales

Orgyia basinigra	Dipterocarpaceae, Fabaceae, Sterculiaceae, Zingiberaceae	Malvales, Fabales, Zingiberales
Orgyia ariadne	Fabaceae	Fabales
Orgyia thyellina	Rosaceae, Moraceae, Fagaceae, Salicaceae, Fabaceae, Cannabaceae, Malvaceae, Platanaceae, Cornaceae, Polygonaceae, Ebenaceae	Rosales, Fagales, Malpighiales, Fabales, Malvales, Proteales, Cornales, Caryophyllales, Ericales
Orgyia truncata	Moraceae	Rosales
Orgyia aurolimbata	Fabaceae	Fabales
Orgyia rupestris	Fabaceae, Plumbaginaceae	Fabales, Caryophyllales
Orgyia corsica	Fagaceae, Fabaceae	Fagales, Fabales
Orgyia affinis	Rubiaceae, Clusiaceae, Smilacaceae	Gentianales, Malpighiales, Liliales
Orgyia antiquoides	Ericaceae, Empetraceae, Rosaceae, Myricaceae, Salicaceae	Ericales, Rosales, Fagales, Malpighiales
Orgyia australis	Mimosaceae, Fabaceae, Myrsinaceae, Araucariaceae, Proteaceae, Theaceae, Geraniaceae, Pinaceae, Sterculiaceae	Fabales, Ericales, Pinales, Proteales, Geraniales, Malvales
Orgyia basinigra	Fabaceae, Lauraceae, Sterculiaceae	Fabales, Laurales, Malvales
Orgyia cana	Rhamnaceae, Rosaceae, Fagaceae, Caprifoliaceae	Rosales, Fagales, Dipsacales
Orgyia tuberculata	Podocarpaceae	Pinales
Orgyia basalis	Fabaceae, Euphorbiaceae, Alliaceae, Rubiaceae, Iridaceae,	Fabales, Malpighiales, Asparagales,

	Flacourtiaceae, Myrtaceae, Geraniaceae, Malvaceae, Myrsinaceae, Pinaceae, Rosaceae,Verbenaceae, Asteraceae, Combretaceae, Sterculiaceae	Gentianales, Myrtales, Geraniales, Malvales, Ericales, Pinales, Rosales, Lamiales, Asterales
Orgyia definita	Aceraceae, Betulaceae, Hamamelidaceae, Salicaceae, Tiliaceae, Ulmaceae	Sapindales, Fagales, Saxifragales, Malpighiales, Malvales, Rosales
Orgyia detrita	Anacardiaceae, Lauraceae, Fagaceae, Taxodiaceae	Sapindales, Laurales, Fagales, Pinales
Orgyia falcata	Fagaceae	Fagales
Orgyia recens	Betulaceae, Ericaceae, Rosaceae, Salicaceae, Fagaceae	Fagales, Ericales, Rosales, Malpighiales
Orgyia pseudotsugata	Pinaceae, Cupressaceae, Celastraceae, Grossulariaceae, Rosaceae, Salicaceae	Pinales, Celastrales, Saxifragales, Rosales, Malpighiales
Orgyia viridescens	Moraceae	Rosales
Orgyia triangularis	Theaceae, Rosaceae, Lythraceae, Fagaceae, Coriariaceae, Santalaceae, Cistaceae	Rosales, Fagales, Ericales, Myrtales, Cucurbitales, Santalales, Malvales
Orgyia hopkinsi	Rubiaceae, Cupressaceae, Myrtaceae, Lythraceae, Pinaceae	Gentianales, Pinales, Myrtales

Orgyia leucostigma	Pinaceae, Aceraceae, Hippocastanaceae, Simaroubaceae, Betulaceae, Rosaceae, Berberidaceae, Juglandaceae, Corylaceae, Bignoniaceae, Fagaceae, Ulmaceae, Rubiaceae, Fabaceae, Asteraceae, Chenopodiaceae, Oleaceae, Ranunculaceae, Convallariaceae, Cornaceae, Anacardiaceae, Cupressaceae, Ebenaceae, Moraceae, Geraniaceae, Theaceae, Malvaceae, Hamamelidaceae, Cannabaceae, Aquifoliaceae, Convolvulaceae, Iridaceae, Ericaceae, Cupressaceae, Poaceae, Magnoliaceae, Caprifoliaceae, Vitaceae, Myricaceae, Scrophulariaceae, Plantaginaceae, Salicaceae, Rhamnaceae, Grossulariaceae, Euphorbiaceae, Lauraceae, Staphyleaceae, Tamaricaceae, Taxaceae, Tiliaceae	Pinales, Sapindales, Fagales, Rosales, Ranunculales, Lamiales, Gentianales, Fabales, Asterales, Caryophyllales, Poales, Asparagales, Cornales, Ericales, Geraniales, Malvales, Saxifragales, Aquifoliales, Solanales, Magnoliales, Dipsacales, Vitales, Malpighiales, Laurales, Crossosomatales
Orgyia dubia	Fabaceae, Salicaceae, Fagaceae, Chenopodiaceae, Asteraceae, Polygonaceae, Amaranthaceae, Ephedraceae, Aizoaceae, Tamaricaceae, Cistaceae	Fabales, Malpighiales, Fagales, Caryophyllales, Asterales, Gnetales, Malvales
Orgyia papuana	Mimosaceae, Verbenaceae, Lauraceae	Fabales, Lamiales, Laurales
Orgyia oslari	Pinaceae	Pinales
Orgyia tricolor	Fabaceae, Aizoaceae	Fabales, Caryophyllales
Orgyia leuschneri	Aceraceae, Fagaceae	Sapindales, Fagales

Orgyia mixta	Malvaceae, Fabaceae, Euphorbiaceae, Begoniaceae, Theaceae, Rutaceae, Casuarinaceae, Rubiaceae, Cupressaceae, Rosaceae, Myrtaceae, Geraniaceae, Lythraceae, Pinaceae, Anacardiaceae, Sterculiaceae, Ulmaceae, Poaceae, Rhamnaceae	Malvales, Fabales, Malpighiales, Cucurbitales, Ericales, Fagales, Sapindales, Gentianales, Pinales, Rosales, Myrtales, Geraniales, Lamiales, Malvales, Poales
Orgyia vetusta	Betulaceae, Asteraceae, Ericaceae, Chenopodiaceae, Fabaceae, Rhamnaceae, Rutaceae, Rosaceae, Polygonaceae, Juglandaceae, Fagaceae, Salicaceae	Fagales, Asterales, Ericales, Caryophyllales, Fabales, Sapindales, Rosales, Malpighiales
Orgyia spp	Arecaceae	Arecales
*Orgyia postica**	Anacardiaceae, Bombacaceae, Casuarinaceae, Combretaceae, Fabaceae, Dipterocarpaceae, Euphorbiaceae, Geraniaceae, Lauraceae, Lythraceae, Myrtaceae, Rhamnaceae, Rosaceae, Rubiaceae, Rutaceae, Santalaceae, Sapindaceae, Sterculiaceae, Theaceae, Tiliaceae, Verbenaceae, Pinaceae, Orchidaceae, Cannaceae, Fagaceae, Boraginaceae, Clusiaceae, Iridaceae, Malpighiaceae, Salicaceae, Tamaricaceae, Cupressaceae, Vitaceae, Ulmaceae, Polygonaceae	Sapindales, Malvales, Fagales, Myrtales, Malpighiales, Geraniales, Laurales, Fabales, Rosales, Gentianales, Malvales, Ericales, Lamiales, Pinales, Asparagales, Zingiberales, Boraginales, Caryophyllales, Vitales, Santalales
*Albarracina warionis**	Ephedraceae	Gnetales

*Bembina isabellina**	Poaceae, Lauraceae	Poales, Laurales
Bembina atripuncta	Sterculiaceae	Malvales
Bembina apicalis	Combretaceae	Myrtales
*Lacida vertiginosa**	Missing	Missing
*Micromorphe linta**	Combretaceae,Rubiaceae	Myrtales, Gentianales
*Euproctis singapura**	Missing	Missing
*Euproctis pyraustis**	Missing	Missing
*Euproctis fumea**	Missing	Missing
*Euproctis seitzi**	Missing	Missing
Euproctis latifascia	Euphorbiaceae, Poaceae, Convolvulaceae, Polygonaceae	Malpighiales, Poales, Solanales, Caryophyllales
Euproctis subfasciata	Poaceae	Poales
Euproctis taiwana	Asteraceae, Iridaceae, Fabaceae, Rubiaceae, Euphorbiaceae, Vitaceae	Asterales, Asparagales, Fabales, Gentianales, Malpighiales, Vitales
Euproctis varians	Theaceae, Rutaceae, Brassicaceae, Euphorbiaceae, Poaceae, Fabaceae, Orchidaceae, Arecaceae, Rhamnaceae	Ericales, Sapindales, Brassicales, Malpighiales, Poales, Fabales, Asparagales, Arecales, Rosales
*Euproctis flavotriangulata**	Juglandaceae	Fagales
*Euproctis callichlaena**	Missing	Missing

*Somena scintillans**	Poaceae, Asteraceae, Malvaceae, Hippocastanaceae, Euphorbiaceae, Amaranthaceae, Oxalidaceae, Berberidaceae, Brassicaceae, Anacardiaceae, Theaceae, Capparaceae, Solanaceae, Fagaceae, Lauraceae, Rutaceae, Verbenaceae, Rubiaceae, Tiliaceae, Coriariaceae, Cucurbitaceae, Myrtaceae, Moraceae, Malvaceae, Sterculiaceae, Lythraceae, Linaceae, Loranthaceae, Rosaceae, Sapindaceae, Salicaceae, Pedaliaceae, Dipterocarpaceae, Combretaceae, Orchidaceae, Rhamnaceae, Elaeocarpaceae, Hamamelidaceae, Fabaceae, Scrophulariaceae, Tamaricaceae, Passifloraceae	Poales, Asterales, Malvales, Sapindales, Malpighiales, Caryophyllales, Oxalidales, Ranunculales, Brassicales, Ericales, Solanales, Fagales, Laurales, Lamiales, Gentianales, Cucurbitales, Myrtales, Rosales, Santalales, Asparagales, Saxifragales, Fabales
Somena kurosawai	Fabaceae, Theaceae, Rutaceae, Rosaceae	Fabales, Ericales, Sapindales, Rosales
Somena pulverea	Theaceae, Rosaceae, Fabaceae	Fabales, Ericales, Rosales
Somena similis	Anacardiaceae, Euphorbiaceae, Fabaceae	Fabales, Sapindales, Malpighiales
*Kidokuga piperita**	Fagaceae, Euphorbiaceae, Theaceae, Betulaceae, Hamamelidaceae, Clethraceae, Styracaceae, Ericaceae, Aceraceae, Fabaceae, Salicaceae, Caprifoliaceae	Fagales, Malpighiales, Ericales, Saxifragales, Sapindales, Fabales, Dipsacales
Kidokuga torasan	Fagaceae	Fagales
*Orvasca subnotata**	Fabaceae, Anacardiaceae, Arecaceae, Cucurbitaceae, Rutaceae,	Fabales, Sapindales, Arecales, Cucurbitales,

	Cycadaceae, Myrtaceae, Euphorbiaceae, Rhizophoraceae, Sapindaceae, Poaceae, Solanaceae, Sterculiaceae, Sonneratiaceae	Cycadales, Myrtales, Malpighiales, Poales, Solanales, Malvales
Orvasca bicolor	Dipterocarpaceae, Euphorbiaceae, Fabaceae	Fabales, Malvales, Malpighiales
Orvasca limbata	Annonaceae, Theaceae, Fabaceae, Melastomataceae, Poaceae	Magnoliales, Ericales, Fabales, Myrtales, Poales
Orvasca aliena	Rosaceae, Mimosaceae, Vitaceae	Rosales, Fabales, Vitales
Orvasca australis	Theaceae, Primulaceae, Fabaceae, Geraniaceae, Mimosaceae, Pinaceae, Proteaceae	Ericales, Fabales, Geraniales, Pinales, Proteales
Orvasca paradoxa	Brassicaceae	Brassicales
Orvasca taiwana	Asteraceae, Brassicaceae, Polygonaceae, Rosaceae	Asterales, Brassicales, Caryophyllales, Rosales
*Sphrageidus similis**	Salicaceae, Fagaceae, Betulaceae, Corylaceae, Rosaceae, Moraceae, Caprifoliaceae, Rhamnaceae, Fabaceae, Paulowniaceae, Ranunculaceae, Rubiaceae, Oleaceae, Polygonaceae, Rutaceae, Juglandaceae, Solanaceae, Poaceae, Grossulariaceae, Amygdaloideae, Adoxaceae, Malvaceae	Malpighiales, Fagales, Rosales, Dipsacales, Fabales, Lamiales, Gentianales, Caryophyllales, Sapindales, Solanales, Poales, Saxifragales, Malvales, Ranunculales
Sphrageidus xanthorrhoea	Asteraceae, Euphorbiaceae, Poaceae, Hamamelidaceae, Fabaceae, Lythraceae, Malvaceae, Rubiaceae, Tamaricaceae, Theaceae	Asterales, Malpighiales, Poales, Saxifragales, Fabales, Myrtales, Malvales, Gentianales, Caryophyllales, Ericales

Sphrageidus virguncula	Anacardiaceae, Brassicaceae, Dioscoreaceae, Euphorbiaceae, Poaceae, Fabaceae, Malvaceae, Nelumbonaceae, Rhamnaceae, Theaceae, Casuarinaceae, Rubiaceae, Tamaricaceae	Sapindales, Brassicales, Dioscoreales, Malvales, Malpighiales, Poales, Fabales, Proteales, Rosales, Ericales, Fagales, Gentianales, Caryophyllales
*Nygmia marginata**	Missing	Missing
*Nygmia quadrangularis**	Missing	Missing
*Nygmia uniformis**	Missing	Missing
*Nygmia javana**	Rubiaceae	Gentianales
*Nygmia plana**	Anacardiaceae, Moraceae, Fagaceae	Sapindales, Rosales, Fagales
*Nygmia staudingeri**	Missing	Missing
Nygmia icilia	Loranthaceae	Santalales
Nygmia xanthomela	Loranthaceae, Dioscoreaceae, Annonaceae, Fabaceae, Rutaceae, Lythraceae, Rosaceae	Santalales, Dioscoreales, Magnoliales, Fabales, Sapindales, Myrtales, Rosales
Nygmia peperites	Moraceae	Rosales
Nygmia solitaria	Loranthaceae, Fabaceae	Santalales, Fabales
Nygmia oreosaura	Sterculiaceae, Apocynaceae, Fabaceae	Malvales, Gentianales, Fabales
Nygmia bimaculata	Juglandaceae, Acanthaceae, Loranthaceae	Fagales, Lamiales, Santalales

Nygmia atereta	Fabaceae	Fabales
Nygmia antiopa	Betulaceae, Salicaceae, Ulmaceae, Urticaceae	Fagales, Malpighiales, Rosales
Nygmia fumosa	Myrtaceae, Euphorbiaceae, Dipterocarpaceae	Myrtales, Malpighiales, Malvales
Nygmia moalata	Amaryllidaceae, Asteraceae, Juglandaceae, Rosaceae, Theaceae	Asparagales, Asterales, Fagales, Rosales, Ericales
Nygmia pelopicta	Moraceae	Rosales
Nygmia corbetti	Euphorbiaceae	Malpighiales
*Arna bipunctapex**	Euphorbiaceae, Theaceae, Cucurbitaceae, Rosaceae, Myrtaceae, Convolvulaceae, Fagaceae, Moraceae, Fabaceae, Lecythidaceae, Salicaceae, Dipterocarpaceae, Combretaceae, Elaeocarpaceae, Ebenaceae, Hamamelidaceae, Lauraceae, Oleaceae, Rosaceae, Rutaceae	Malpighiales, Ericales, Cucurbitales, Rosales, Myrtales, Solanales, Fagales, Fabales, Malvales, Oxalidales, Saxifragales, Laurales, Lamiales, Sapindales
*Arna bicostata**	Missing	Missing
Arna pseudoconspersa	Theaceae, Rosaceae, Poaceae, Euphorbiaceae, Rutaceae, Ebenaceae	Ericales, Rosales, Malpighiales, Sapindales, Poales
Arna perplexa	Rubiaceae	Gentianales
Arna phaulida	Combretaceae	Myrtales
*Artaxa rubiginosa**	Missing	Missing

*Euproctis cryptosticta**	Euphorbiaceae, Smilacaceae	Malpighiales, Liliales
*Euproctis kanshireia**	Fagaceae, Rosaceae, Rutaceae, Myrsinaceae, Euphorbiaceae	Fagales, Rosales, Sapindales, Ericales, Malpighiales
*Artaxa angulata**	Fabaceae	Fabales
*Artaxa digramma**	Melastomataceae, Anacardiaceae, Combretaceae, Dipterocarpaceae, Lythraceae, Sapindaceae, Rosaceae, Polygonaceae	Myrtales, Sapindales, Malvales, Caryophyllales Sapindales, Rosales
Artaxa sakaguchii	Fagaceae	Fagales
Artaxa subflava	Rosaceae, Ebenaceae, Polygonaceae, Ericaceae, Urticaceae, Theaceae, Euphorbiaceae, Lythraceae, Malvaceae, Rhamnaceae, Rubiaceae	Rosales, Ericales, Caryophyllales, Malpighiales, Myrtales, Malvales, Gentianales
Artaxa guttata	Anacardiaceae, Apocynaceae, Combretaceae, Dipterocarpaceae, Euphorbiaceae, Lecythidaceae, Fabaceae, Lythraceae, Myrsinaceae, Oleaceae, Rhamnaceae, Verbenaceae	Sapindales, Gentianales, Myrtales, Malvales, Malpighiales, Ericales, Fabales, Lamiales, Rosales
Artaxa pauperata	Poaceae	Poales
Artaxa ormea	Euphorbiaceae	Malpighiales
Artaxa flava	Rosaceae, Ebenaceae, Fagaceae, Punicaceae, Aceraceae, Betulaceae, Cupressaceae, Pinaceae, Anacardiaceae, Euphorbiaceae, Combretaceae, Rubiaceae, Solanaceae	Rosales, Ericales, Fagales, Myrtales, Sapindales, Pinales, Malpighiales, Gentianales, Solanales

*Toxoproctis croceola**	Juglandaceae	Fagales
Toxoproctis cincta	Nelumbonaceae	Proteales
Toxoproctis munda	Annonaceae, Rubiaceae	Magnoliales, Gentianales
Toxoproctis flavociliata	Euphorbiaceae	Malpighiales
Toxoproctis bifurcata	Melastomataceae	Myrtales
Euproctis montis	Rosaceae, Vitaceae, Rutaceae, Moraceae, Theaceae, Solanaceae	Rosales, Vitales, Sapindales, Ericales, Solanales
*Euproctis collenettei**	Missing	Missing
*Euproctis ?aureoplaga**	Missing	Missing
*Euproctis molunduana**	Missing	Missing
*Euproctis ionthana**	Missing	Missing
*Euproctis ?putris**	Missing	Missing
*Euproctis chrysorrhoea**	Rosaceae, Fabaceae, Aceraceae, Asteraceae, Betulaceae, Juglandaceae, Fagaceae, Oleaceae, Geraniaceae, Elaeagnaceae, Myricaceae,Vitaceae, Plantaginaceae, Salicaceae, Polygonaceae, Grossulariaceae, Caprifoliaceae, Tiliaceae, Ulmaceae, Rutaceae, Hippocastanaceae	Rosales, Fabales, Sapindales, Asterales, Fagales, Geraniales, Vitales, Lamiales, Malpighiales, Caryophyllales, Saxifragales, Dipsacales, Malvales
*Euproctis karghalica**	Salicaceae, Elaeagnaceae, Moraceae, Rosaceae	Rosales, Malpighiales

*Euproctis conistica**	Missing	Missing
*Euproctis yunnanpina**	Pinaceae	Pinales
*Euproctis magna**	Missing	Missing
*Nygmia inornata**	Euphorbiaceae	Malpighiales
*Euproctis ?serrula**	Missing	Missing

Note: “*” refers to the taxa occurring in the Lymantriinae phylogeny of Wang et al. (2015).
