# Supplementary material for: Polyphagy and diversification in tussock moths: Support for the oscillation hypothesis from extreme generalists
Source: Ecol Evol. 2017 Aug 30;7(19):7975–86. doi: 10.1002/ece3.3350 (PMC5632610; doi:10.1002/ece3.3350)
Supplement: Supplementary file 3 [file ECE3-7-7975-s003.pdf]

Table S2. Species numbers of Lymantriinae genus-level taxa and the number of host plant orders used.

Genus names (Lymantriinae)	Species numbers (Lymantriinae)	Order numbers (Host plants)
Daplasa	5	unknown
Arctornis	155	14
Ruanda	3	1
Eloria	70	5
Euproctoides	5	unknown
Locharna	5	5
Kuromondokuga	4	4
Pida	13	1
Leucoma	46	12
"Leucoma" sericea	1	2
Perina	6	3
"Leucoma" chrysoscela	1	unknown
Ivela	3	4
Olapa	18	4
Caviria	13	8
Thagona	39	6
Imaus	2	unknown
Cispia	13	2
Dura	25	unknown
Crorema	17	2
Sarsina	7	3

Lymantria	170	34
Aroa	18	5
Hemerophanes	6	1
Ilema	30	16
Mylantria	1	4
Griveaudyria	2	1
Calliteara	45	28
Laelia	100	6
Pantana	33	1
Neomardara	2	unknown
"Olene" & "Telochurus"	11	5
Cifuna	5	9
Dasychira	16	10
Psalis	11	11
Teia	18	21
Olene	26	25
Orgyia	62	38
Albaraccina	2	1
Bembina	6	4
Lacida	7	unknown
Micromorphe	6	2
"Euproctis"1	20	14
"Euproctis"2	1	1
"Euproctis"3	1	unknown
Somena	6	22
Kidokuga	2	7

Orvasca	28	20
Sphrageidus	3	20
Nygmia	55	15
Arna	14	16
Artaxa	33	15
Toxoproctis	30	11
Euproctis	9	14

Table S3. Most polyphagous species in each contrast in Table 2

Sister pairing	MP1	HR1	MP2	HR2
Somena-Kidokuga	*S. scintillans*	22	*K. piperita*	7
(Arna+Artaxa+Toxoproctis+Euproctis)-Nygmia	*Arna bipunctapex*	14	*N. xanthomela*	7
Calliteara-Griveaudyria	*C. horsfieldi*	18	*G. ila*	1
Laelia-Pantana	*L. coenosa*	4	*P. substrigosa* etc.	1
Orgyia-Olene	*O. leucostigma*	25	*O. mendosa*	20
Aroa-Hemerophanes	*A. difficilis* etc.	2	*H. enos* etc.	1
Lymantria-(Sarsina+Crorema)	*L.dispar*	25	*S. violascens*	3
Leucoma-(Ivela+Perina+?Leucoma)	*L. salicis*	6	*P. nuda*	3
(Locharna+Kuromondokuga)-Pida	*L. strigipennis* etc.	4	*Pida spp.*	1
Eloria-Ruanda	*E. torrida* etc.	3	*R. nuda*	1

Notes: MP1 = Most polyphagous species in clade with highest total host diversity, with its host range HR1 (number of orders);

MP2 = Most polyphagous species in clade with lowest host diversity, with its host range HR2
