# Supplementary material for: Polyphagy and diversification in tussock moths: Support for the oscillation hypothesis from extreme generalists
Source: Ecol Evol. 2017 Aug 30;7(19):7975–86. doi: 10.1002/ece3.3350 (PMC5632610; doi:10.1002/ece3.3350)
Supplement: Supplementary file 4 [file ECE3-7-7975-s004.pdf]

Table S4. Host use in Lymantriinae and its closest relatives

Subfamily	Genus	Total orders	Max pol	#records	#3+	% 3+	#7+	% 7+
Lymantriinae	*Arctornis*	14	5	27	3	11%	0	0%
	Ruanda	1	1	1	0	0%	0	0%
	Eloria	5	3	8	2	25%	0	0%
	Locharna	5	4	2	1	50%	0	0%
	Kuromondokuga	4	4	2	1	50%	0	0%
	Pida	1	1	1	0	0%	0	0%
	Leucoma	12	6	8	3	38%	0	0%
	"Leucoma" sericea	2	2	1	0	0%	0	0%
	Perina	3	3	2	1	50%	0	0%
	Ivela	4	2	3	0	0%	0	0%
	Olapa	4	2	5	0	0%	0	0%
	Caviria	8	4	1	1	100%	0	0%
	Thagona	6	4	4	1	25%	0	0%
	Cispia	2	1	4	0	0%	0	0%
	Crorema	2	1	2	0	0%	0	0%
	Sarsina	3	3	3	1	33%	0	0%
	Lymantria	34	25	53	18	34%	7	13%
	Aroa	5	2	5	0	0%	0	0%
	Hemerophanes	1	1	2	0	0%	0	0%
	Ilema	16	8	9	5	56%	1	11%
	Mylantria	4	4	1	1	100%	0	0%
	Griveaudyria	1	1	1	0	0%	0	0%
	Calliteara	28	18	23	7	30%	4	17%

	Laelia	6	4	16	1	6%	0	0%
	Pantana	1	1	10	0	0%	0	0%
	"Olene"/ "Telochurus"	5	2	2	0	0%	0	0%
	Cifuna	9	9	1	1	100%	1	100%
	Dasychira	10	7	14	8	57%	1	7%
	Psalis	11	11	1	1	100%	1	100%
	Teia	21	17	7	4	57%	1	14%
	Olene	25	20	2	2	100%	2	100%
	Orgyia	38	25	36	24	67%	10	28%
	Albarracina	1	1	1	0	0%	0	0%
	Bembina	4	2	3	0	0%	0	0%
	Micromorphe	2	2	1	0	0%	0	0%
	Euproctis_1	14	9	4	3	75%	1	25%
	Euproctis_2	1	1	1	0	0%	0	0%
	Somena	22	22	4	4	100%	1	25%
	Kidokuga	7	7	2	1	50%	1	50%
	Orvasca	20	10	7	6	86%	1	14%
	Sphrageidus	20	14	3	3	100%	3	100%
	Nygmia	15	7	14	7	50%	1	7%
	Arna	16	14	4	2	50%	1	25%
	Artaxa	15	9	10	5	50%	3	30%
	Toxoproctis	11	5	6	1	17%	0	0%
	Euproctis	14	13	4	1	25%	1	25%
Hypeninae	*Colobochyla*	1	1	2	0	0%	0	0%
	Hypena	18	11	76	6	8%	1	1%

Pangraptinae	*Episparis*	3	2	3	0	0%	0	0%
	Gracilodes	2	1	2	0	0%	0	0%
	Pangrapta	6	1	13	0	0%	0	0%
Herminiinae	*Idia*	7	3	5	1	20%	0	0%
	Paracolax	2	2	6	0	0%	0	0%
	Polypogon	6	4	3	1	33%	0	0%
	Herminia	9	5	5	2	40%	0	0%
	Simplicia	17	8	10	5	50%	1	10%
	Nodaria	5	3	3	1	33%	0	0%
Aganainae	*Mecodina*	4	2	5	0	0%	0	0%
	Psimada	1	1	1	0	0%	0	0%
	Neochera	1	1	1	0	0%	0	0%
	Asota	12	11	15	2	13%	1	7%
	Peridrome	1	1	1	0	0%	0	0%
Arctiinae	*Cyana*	7	5	3	1	33%	0	0%
	Amerila	14	6	12	3	25%	0	0%
	Dysauxes	2	2	2	0	0%	0	0%
	Apisa	1	1	1	0	0%	0	0%
	Syntomis (Amata)	23	8	23	11	48%	2	9%
	Ctenucha	5	2	5	0	0%	0	0%
	Antichloris	2	2	3	0	0%	0	0%
	Coscinia	5	4	3	2	67%	0	0%
	Pseudophaloe	1	1	1	0	0%	0	0%
	Dysschema	19	13	18	6	33%	3	17%
	Callimorpha	9	8	4	2	50%	1	25%
	Nyctemera	13	4	16	3	19%	0	0%

Creatonotos	20	20	6	4	67%	2	33%
Arctia	20	18	4	3	75%	1	25%
Lymantriinae (Average)	9.8	6.9			37%		15%
Related subfamilies (Average)	7.9	5.0			20%		4%
t-test	p=0.32	p=0.20			p<0.05		p=0.05

Notes: Total number of host orders used in each genus, followed by: Max pol = number of orders used by most polyphagous species; #records = number of species with host records; #3+ and %3+ = number and percentage of species out of these feeding on at least three orders; #7+ and %7+ = respectively for feeding on at least seven host orders. T-tests compare levels of polyphagy in Lymantriinae with related subfamilies.

Table S5. Feeding habits of adults and flight abilities of female adults in selected erebid moths

Subfamily	Tribe	Genus	Feeding/Nonfeeding	Female flight abilities
unassigned	unassigned	*Cultripalpa*	Feeding	Flight
unassigned	unassigned	*Colobochyla*	Feeding	flight
Hypeninae	unassigned	*Hypena*	Feeding	flight
Pangraptinae	unassigned	*Masca*	Feeding	Flight
Pangraptinae	unassigned	*Epidparis*	Feeding	Flight
Pangraptinae	unassigned	*Gracilodes*	Feeding	Flight
Pangraptinae	unassigned	*Hyposemansis*	Feeding	Flight
Pangraptinae	unassigned	*Chrysograpta*	Feeding	Flight
Pangraptinae	unassigned	*Pangrapta*	Feeding	Flight
unassigned	unassigned	*Schistorhynx*	Feeding	Flight
Herminiinae	unassigned	*Idia*	feeding	Flight
Herminiinae	unassigned	*Paracolax*	Feeding	Flight
Herminiinae	unassigned	*Polypogon*	Feeding	Flight
Herminiinae	unassigned	*Herminia*	Feeding	Flight
Herminiinae	unassigned	*Lysimelia*	Feeding	Flight
Herminiinae	unassigned	*Simplicia*	Feeding	Flight
Herminiinae	unassigned	*Nodaria*	Feeding	Flight
Aganainae	unassigned	*Mecodina*	Feeding	Flight
Aganainae	unassigned	*Psimada*	Feeding	Flight
Aganainae	Aganaini	*Neochera*	Feeding	Flight
Aganainae	Aganaini	*Asota*	Feeding	Flight
Aganainae	Aganaini	*Euplocia*	Feeding	Flight
Aganainae	Aganaini	*Peridrome*	Feeding	Flight
Arctiinae	Lithosiini	*Garudinia*	Unknown	Flight

Arctiinae	Lithosiini	*Eugoa*	Unknown	Flight
Arctiinae	Lithosiini	*Brunia*	Unknown	Flight
Arctiinae	Lithosiini	*Cyana*	Unknown	Flight
Arctiinae	Lithosiini	*Barsine*	Unknown	Flight
Arctiinae	Amerilini	*Amerila*	Feeding/nonfeeding	Flight
Arctiinae	Syntomini	*Dysauxes*	Unknown	Flight
Arctiinae	Syntomini	*Apisa*	Unknown	Flight
Arctiinae	Syntomini	*Syntomis*	Unknown	Flightless
Arctiinae	Arctiini	*Ctenucha*	Unknown	Flight
Arctiinae	Arctiini	*Antichloris*	Unknown	Flight
Arctiinae	Arctiini	*Coscinia*	Unknown	Flightless
Arctiinae	Arctiini	*Pseudophaloe*	Unknown	Flight
Arctiinae	Arctiini	*Dysschema*	Nonfeeding	Flight
Arctiinae	Arctiini	*Callimorpha*	Unknown	Flight
Arctiinae	Arctiini	*Nyctemera*	Feeding	Flight
Arctiinae	Arctiini	*Creatonotos*	Nonfeeding	Flight
Arctiinae	Arctiini	*Arctia*	Unknown	Flight
Lymantriinae	Daplasini	*Daplasa*	Nonfeeding	Flight
Lymantriinae	Arctornithini	*Arctornis* (incl.*"Sitvia", "Topemesoides", "Carriola"*)	Nonfeeding	Flight
Lymantriinae	Locharnini	*Ruanda*	Nonfeeding	Flight
Lymantriinae	Locharnini	*Eloria*	Nonfeeding	Flight
Lymantriinae	Locharnini	*Euproctoides*	Nonfeeding	Flight
Lymantriinae	Locharnini	*Locharna*	Nonfeeding	Flight
Lymantriinae	Locharnini	*Kuromondokuga*	Nonfeeding	Flight

Lymantriinae	Locharnini	*Pida*	Nonfeeding	Flight
Lymantriinae	Leucomini	*Leucoma*	Nonfeeding	Flight
Lymantriinae	Leucomini	*"Leucoma" sericea*	Nonfeeding	Flight
Lymantriinae	Leucomini	*Perina*	Nonfeeding	Flight
Lymantriinae	Leucomini	*Ivela*	Nonfeeding	Flight
Lymantriinae	Lymantriini	*Olapa*	Nonfeeding	Flight
Lymantriinae	Lymantriini	*Caviria*	Nonfeeding	Flight
Lymantriinae	Lymantriini	*Thagona*	Nonfeeding	Flight
Lymantriinae	Lymantriini	*Imaus*	Nonfeeding	Flight
Lymantriinae	Lymantriini	*Cispia*	Nonfeeding	Flight
Lymantriinae	Lymantriini	*Dura*	Nonfeeding	Flight
Lymantriinae	Lymantriini	*Crorema*	Nonfeeding	Flight
Lymantriinae	Lymantriini	*Sarsina*	Nonfeeding	Flight
Lymantriinae	Lymantriini	*Lymantria*	Nonfeeding	Flightless
Lymantriinae	Orgyiini	*Aroa*	Nonfeeding	Flightless
Lymantriinae	Orgyiini	*Hemerophanes*	Nonfeeding	Flight
Lymantriinae	Orgyiini	*Ilema*	Nonfeeding	Flight
Lymantriinae	Orgyiini	*Mylantria*	Nonfeeding	Flight
Lymantriinae	Orgyiini	*Griveaudyria*	Nonfeeding	Flight
Lymantriinae	Orgyiini	*Calliteara*	Nonfeeding	Flight
Lymantriinae	Orgyiini	*Laelia*	Nonfeeding	Flightless
Lymantriinae	Orgyiini	*Pantana*	Nonfeeding	Flight
Lymantriinae	Orgyiini	*Neomardara*	Nonfeeding	Flight
Lymantriinae	Orgyiini	*Telochurus*	Nonfeeding	Flightless
Lymantriinae	Orgyiini	*Cifuna*	Nonfeeding	Unkown
Lymantriinae	Orgyiini	*Dasychira*	Nonfeeding	Flight
Lymantriinae	Orgyiini	*Psalis*	Nonfeeding	Flight

Lymantriinae	Orgyiini	*Teia*	Nonfeeding	Flightless
Lymantriinae	Orgyiini	*Olene*	Nonfeeding	Flight & fightless
Lymantriinae	Orgyiini	*Orgyia*	Nonfeeding	Flightless
Lymantriinae	Nygmiini	*Albarracina*	Nonfeeding	Flight
Lymantriinae	Nygmiini	*Bembina*	Nonfeeding	Flight
Lymantriinae	Nygmiini	*Lacida*	Nonfeeding	Flight
Lymantriinae	Nygmiini	*Micromorphe*	Nonfeeding	Flight
Lymantriinae	Nygmiini	*Euproctis*_1	Nonfeeding	Flight
Lymantriinae	Nygmiini	*Euproctis*_2	Nonfeeding	Flight
Lymantriinae	Nygmiini	*Euproctis*_3	Nonfeeding	Flight
Lymantriinae	Nygmiini	*Somena*	Nonfeeding	Flight
Lymantriinae	Nygmiini	*Kidokuga*	Nonfeeding	Flight
Lymantriinae	Nygmiini	*Orvasca*	Nonfeeding	Flight
Lymantriinae	Nygmiini	*Sphrageidus*	Nonfeeding	Flight
Lymantriinae	Nygmiini	*Nygmia*	Nonfeeding	Flight
Lymantriinae	Nygmiini	*Arna*	Nonfeeding	Flight
Lymantriinae	Nygmiini	*Artaxa*	Nonfeeding	Flight
Lymantriinae	Nygmiini	*Toxoproctis*	Nonfeeding	Flight
Lymantriinae	Nygmiini	*Euproctis*	Nonfeeding	Flight

Table S6. Host plant use of Erebid taxa in the oriental tropics

Subfamilies (Erebidae)	Tribes (Erebidae)	Subtribes (Erebidae)	Numbers of host plant genera with the plant family names
Aganainae	-	-	18 Moraceae; 6 Apocynaceae; 2 Meliaceae; 1 Asclepiadacae, Asteraceae, Caricaceae, Clusiaceae, Dipterocarpaceae, Euphorbiaceae, Fabaceae, Myrtaceae, Oxalidaceae, Rubiaceae, Solanaceae, Theaceae, Verbenaceae
Pangraptinae	-	-	12 Rubiaceae; 2 Oleaceae; 1 Magnoliaceae, Meliaceae, Sapindaceae, Smilacaceae
Calpinae	-	-	42 Menispermaceae; 3 Sapindaceae; 1 Amaranthaceae, Clusiaceae, Fabaceae, Lardizabalaceae, Sterculiaceae
Scoliopterygina e	-	-	37 Malvaceae*; 7 Sterculiaceae*; 5 Fabaceae; 3 Bombacaceae*, Tiliaceae*; 2 Euphorbiaceae; 1 Amaranthaceae, Convolvulaceae, Menispermaceae; Moraceae, Rosaceae, Rubiaceae, Solanaceae, Verbenaceae
Erebinae	Hulodini	-	19 Fabaceae; 2 Nyctaginaceae, Rubiaceae; 1 Acanthaceae, Adiantaceae, Apocynaceae, Dipterocarpaceae, Lythraceae, Rutaceae, Theaceae
Erebinae	Hypopyrini	-	7 Fabaceae; 1 Theaceae, Vitaceae

Erebinae	Pandesmini	-	5 Fabaceae; 1 Salicaceae, Sapindaceae
Erebinae	Pericymini	-	9 Fabaceae; 1 Rubiaceae, Sapindaceae
Erebinae	Erebini	-	8 Fabaceae; 2 Smilacaceae; 1 Malvaceae
Erebinae	Euclidiini	-	23 Fabaceae; 11 Poaceae; 2 Sapindaceae; 1 Araceae, Cyperaceae, Dipterocarpaceae, Euphorbiaceae, Malvaceae, Rubiaceae, Solanaceae, Zingiberaceae
Erebinae	Cocytiini	-	16 Sapindaceae; 1 Arecaceae, Tiliaceae, Verbenaceae, Vitaceae
Erebinae	Ophiusini	-	12 Combretaceae; 4 Myrtaceae; 2 Anacardiaceae, Dipterocarpaceae, Lecythidaceae, Pinaceae, Rosaceae, Vitaceae; 1 Euphorbiaceae, Fagaceae, Geraniaceae, Lauraceae, Menispermaceae, Sapindaceae, Verbenaceae
Erebinae	Poaphilini	-	30 Euphorbiaceae; 14 Fabaceae; 9 Sapotaceae; 6 Sapindaceae; 4 Rhamnaceae; 3 Rubiaceae; 2 Araucariaceae, Brassicaceae, Celastraceae, Convolvulaceae, Coriariaceae, Poaceae, Rosaceae, Rutaceae, Sterculiaceae, Tamaricaceae; 1 Anacardiaceae, Cucurbitaceae, Cupressaceae, Dipterocarpaceae, Ebenaceae, Geraniaceae, Lecythidaceae, Linaceae, Loganiaceae, Lythraceae, Meliaceae, Moraceae, Polypodiaceae, Polygonaceae, Proteaceae, Solanaceae, Tiliaceae, Vitaceae, Zygophyllaceae.
Erebidae	Ommatophorin	-	1 Fabaceae

i

Erebidae	Acantholipini	-	2 Fabaceae, 1 Connaraceae
Erebidae	Catephiini	-	1 Combretaceae, Lythraceae
Erebidae	Catocalini	-	1 Fagaceae, Oleaceae, Solanaceae
Erebidae	Sypnini	-	2 Rosaceae; 1 Fagaceae
Erebidae	Ercheiini	-	2 Poaceae; 1 Asparagaceae, Brassicaceae, Fabaceae, Poaceae, Tiliaceae
Erebidae	Amphigoniini	-	No records
Anobinae	-	-	16 Fabaceae; 1 Poaceae
Eulepidotinae	-	-	8 Fabaceae; 4 Poaceae; 1 Cucurbitaceae
Hypocallinae	-	-	7 Ebenaceae; 3 Fagaceae; 2 Fabaceae, Meliaceae, Rutaceae; 1 Anacardiaceae, Euphorbiaceae, Lecythidaceae, Sapindaceae, Sapotaceae, Clusiaceae, Rosaceae
Tinoliinae	-	-	12 Acanthaceae; 1 Rhamnaceae
Rivulinae	-	-	5 Poaceae; 2 Marantaceae; 1 Anacardiaceae
Hypeninae	-	-	16 Fabaceae; 7 Urticaceae; 6 Verbenaceae; 4 Acanthaceae; 2 Asclepiadaceae, Cannabaceae, Connaraceae; 1 Anacardiaceae, Combretaceae, Lamiaceae, Poaceae, Polygonaceae, Rutaceae
Arctiinae	Syntomini	-	5 Asteraceae; 4 Fabaceae; 3 Poaceae; 2 Anacardiaceae, Theaceae; 1 Brassicaceae,

			Convolvulaceae, Cucurbitaceae, Fagaceae, Rutaceae, Santalaceae, Solanaceae, Theaceae, Verbenaceae, Vitaceae
Arctiinae	Amerilini	-	1 Apocynaceae, Asclepiadaceae, Dioscoreaceae, Rubiaceae, Smilacaceae
Arctiinae	Arctiini	Callimorphinae	13 Fabaceae; 12 Boraginaceae; 1 Alliaceae, Asteraceae, Musaceae, Poaceae, Sterculiaceae, Verbenaceae
Arctiinae	Arctiini	Pericopinae	18 Asteraceae; 1 Brassicaceae, Fabaceae, Orchidaceae, Rubiaceae
Arctiinae	Arctiini	Euchromiina	3 Convolvulaceae; 1 Dioscoreaceae, Malvaceae, Passifloraceae, Scrophulariaceae
Arctiinae	Arctiini	Spilosomina	30 Fabaceae; 21 Poaceae; 16 Asteraceae; 8 Verbenaceae; 6 Malvaceae, Rubiaceae, Solanaceae; 5 Convolvulaceae, Meliaceae; 4 Dioscoreaceae, Moraceae; 3 Lamiaceae, Orchidaceae, Pedaliaceae; 2 Arecaceae, Boraginaceae, Brassicaceae, Chenopodiacae, Cucurbitaceae, Euphorbiaceae, Musaceae, Scrophulariaceae, Urticaceae; 1 Acanthaceae, Amaranthaceae, Anacardiaceae, Asparagaceae, Bombacaceae, Cannabaceae, Caprifoliaceae, Commelinaceae, Geraniaceae, Goodeniaceae, Linaceae, Lythraceae, Magnoliacease, Plantaginaceae, Polygonaceae, Rosaceae, Salicaceae, Sterculiaceae, Tiliaceae, Zingiberaceae
Lymantriinae	Lymantriini	-	15 Fabaceae; 13 Dipterocarpaceae; 10 Combretaceae; 9 Anacardiaceae, Rosaceae; 8 Myrtaceae; 7 Euphorbiaceae, Lythraceae; 6 Fagaceae, Rubiaceae; 5 Pinaceae; 4 Casuarinaceae, Moraceae; 3

			Lecythidaceae; 2 Arecaceae, Hamamelidaceae, Meliaceae, Salicaceae, Sterculiaceae, Theaceae, Verbenaceae; 1 Apocynaceae, Begoniaceae, Betulaceae, Bombacaceae, Ebenaceae, Geraniaceae, Juglandaceae, Loranthaceae, Malvaceae, Rhamnaceae, Rutaceae, Sapotaceae, Turneraceae
Lymantriinae	Orgyiini	-	87 Fabaceae; 28 Poaceae; 19 Myrtaceae, Rosaceae; 18 Dipterocarpaceae; 16 Euphorbiaceae; 13 Rubiaceae; 11 Anacardiaceae, Sapindaceae; 10 Verbenaceae; 9 Bombacaceae, Moraceae; 8 Lauraceae, Sterculiaceae; 7 Pinaceae, Theaceae; 6 Arecaceae, Combretaceae, Fagaceae, Lythraceae; 5 Lecythidaceae, Malvaceae, Sapotaceae; 4 Brassicaceae, Geraniaceae, Rutaceae, Salicaceae, Solanaceae, Tiliaceae; 3 Asteraceae, Cannaceae, Clusiaceae, Ericaceae, Melastomataceae, Rhamnaceae; 2 Annonaceae, Apocynaceae, Cannabaceae, Loranthaceae, Musaceae, Orchidaceae, Oxalidaceae, Polygonaceae, Vitaceae; 1 Acanthaceae, Asparagaceae, Begoniaceae, Berberidaceae, Betulaceae, Bixaceae, Boraginaceae, Convolvulaceae, Corylaceae, Cucurbitaceae, Cupressaceae, Cyperaceae, Dilleniaceae, Dioscoreaceae, Ebenaceae, Gleicheniaceae, Iridaceae, Lamiaceae, Leeaceae, Linaceae, Malpighiaceae, Marantaceae, Moringaceae, Nelumbonaceae, Proteaceae, Santalaceae, Tamaricaceae, Urticaceae
Lymantriinae	Daplasini	-	No records
Lymantriinae	Locharnini	-	1 Leeaceae

Lymantriinae	Arctornithini	-	10 Dipterocarpaceae; 5 Lauraceae; 4 Anacardiaceae, Theaceae; 3 Bombacaceae, Combretaceae; 2 Sapindaceae, Sterculiaceae; 1 Brassicaceae, Ebenaceae, Euphorbiaceae, Lythraceae, Poaceae
Lymantriinae	Leucomini	-	7 Lauraceae; 4 Moraceae; 2 Euphorbiaceae, Salicaceae, Ulmaceae; 1 Acanthaceae, Anacardiaceae, Bombacaceae, Magnoliaceae, Sapindaceae, Theaceae
Lymantriinae	Nygmiini	-	78 Fabaceae; 36 Euphorbiaceae; 34 Poaceae; 22 Anacardiaceae; 15 Myrtaceae; 14 Combretaceae, Theaceae; 13 Rubiaceae; 11 Lauraceae; 10 Dipterocarpaceae, Lythraceae, Malvaceae, Rutaceae; 9 Rhamnaceae; 8 Loranthaceae; 7 Asteraceae, Moraceae; 6 Melastomataceae, Sapindaceae; 5 Fagaceae; 4 Apocynaceae, Arecaceae, Brassicaceae, Cucurbitaceae, Sterculiaceae, Tamaricaceae, Verbenaceae; 3 Lecythidaceae, Nelumbonaceae, Orchidaceae, Salicaceae, Solanaceae; 2 Annonaceae, Asclepiadaceae, Convolvulaceae, Coriariaceae, Juglandaceae, Leeaceae, Rhizophoraceae, Sapotaceae, Tiliaceae, Zingiberaceae; 1 Acanthaceae, Acoraceae, Amaranthaceae, Amaryllidaceae, Berberidaceae, Bombacaceae, Capparaceae, Casuarinaceae, Cycadaceae, Geraniaceae, Hamamelidaceae, Hippocastanaceae, Iridaceae, Linaceae, Magnoliaceae, Musaceae, Meliaceae, Myrsinaceae, Oleaceae, Oxalidaceae, Passifloraceae, Pedaliaceae, Plantaginaceae, Tropaeolaceae, Vitaceae

Notes: The host plant data of this table are collected mainly from Robinson et al. (2001), with a few from Common (1990) and Holloway (2005, 2011). Families with asterisks may now be absorbed into Malvaceae in APG system (Angiosperm Phylogeny Group system).
