# Supplementary material for: Polyphagy and diversification in tussock moths: Support for the oscillation hypothesis from extreme generalists
Source: Ecol Evol. 2017 Aug 30;7(19):7975–86. doi: 10.1002/ece3.3350 (PMC5632610; doi:10.1002/ece3.3350)
Supplement: Supplementary file 5 [file ECE3-7-7975-s005.pdf]

Table S7. Species numbers of Lymantriinae genus-level taxa, restricted to well-studied regional faunas, and the number of host plant orders used globally in these clades. Analyses were performed with and without some problematic taxa (in bold).

Genus names	Order numbers (Host plants)	Species numbers
Arctornis	12	88
Leucoma	7	6
Lymantria	31	34
Albarracina	1	1
Sphrageidus	18	2
Laelia	4	5
Calliteara	23	17
Euproctis "chrysorrhoea"	**13**	**1**
Orgyia	35	23
Telochurus	5	2
Dasychira	10	16
Olene "mendosa"	**24**	**2**
Olene "dudgeoni"	**2**	**1**
Ilema	14	10
Arna	16	8
Somena	5	4
Orvasca	14	14
Artaxa	9	16
Micromorphe	2	4
Toxoproctis	5	18
Nygmia "javana"	**13**	**36**
Bembina	2	3
Cispia	1	1
Locharna	1	1
Kidokuga	7	2

Perina	3	2
Kuromondokuga	4	3
Ivela	3	2
Cifuna	9	1
Pantana	1	3
Lacida	Missing	1
Imaus	Missing	1
Dura	Missing	2
Daplasa	Missing	2
